# Extending Carrier Diffusion via Interfacial Dielectric Shielding for Operationally Stable Perovskite/TOPCon Tandem Solar Cells

**DOI:** 10.1002/advs.202524128

**Published:** 2026-01-28

**Authors:** Wenfeng Liu, Zhiqin Ying, Huan Li, Xin Li, Haofan Ma, Yunyun Yu, Ziyu He, Rui Li, Meili Zhang, Yuheng Zeng, Luyao Zheng, Xi Yang, Jichun Ye

**Affiliations:** ^1^ School of Energy Science and Engineering Central South University Changsha China; ^2^ Ningbo Institute of Materials Technology and Engineering Chinese Academy of Sciences (CAS) Ningbo China; ^3^ Yongjiang Laboratory Ningbo Zhejiang China

**Keywords:** carrier accumulation, carrier diffusion length, high dielectric oxide, long‐term stability, perovskite/silicon tandem solar cells

## Abstract

Perovskite/silicon tandem solar cells have achieved remarkable power conversion efficiencies beyond the Shockley–Queisser limit of single‐junction devices. However, their operational stability remains a key challenge, with the low‐dielectric perovskite/C_60_ contact—though often negligible in single‐junction devices—being one of several factors that limit carrier diffusion length and thereby aggravate carrier accumulation and recombination in the thick perovskite layer required for textured silicon in tandems. To address this, a high‐dielectric‐constant niobium oxide (NbO_X_) electron‐selective contact is introduced, which suppresses defect‐mediated carrier trapping by shrinking the capture radius of interfacial defects and chemically passivates undercoordinated Pb^2+^ and PbI_2_ through Pb–O bond formation. These effects collectively extend the carrier diffusion length and optimize energy‐level alignment at the perovskite/C_60_ interface. As a result, single‐junction 1.68 eV perovskite solar cells deliver a PCE of 22.4% with 91% efficiency retention after 650 h of maximum power point (MPP) tracking, while monolithic perovskite/TOPCon tandems reach 32.0% PCE and maintain full initial performance after 200 h of MPP tracking. These results underscore the vital role of extended carrier diffusion in achieving both high efficiency and Carrier accumulation. High dielectric oxide, Perovskite/silicon tandem solar cells, carrier diffusion length, long‐term stability, and long‐term operational stability in perovskite/silicon tandems.

## Graphical abstract

A high‐dielectric niobium oxide (NbO_X_) electron‐selective contact is introduced to modulate the perovskite/C_60_ interface in perovskite/silicon tandem solar cells. By shrinking the defect capture radius and chemically passivating Pb‐related defects, NbO_X_ extends carrier diffusion length and optimizes energy‐level alignment, enabling both high efficiency and enhanced operational stability in single‐junction and tandem devices.

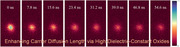

## 1 Introduction

Perovskite/silicon tandem solar cells have recently achieved certified power conversion efficiencies (PCEs) approaching 35% [1], far exceeding the Shockley–Queisser limit of single‐junction solar cells and establishing them as a leading platform for next‐generation high‐efficiency photovoltaics [2]. However, despite these impressive advances in efficiency, their operational stability still falls far short of that of crystalline silicon solar cells [3]. One of the primary origins of this instability lies at the top perovskite/electron transport layer (ETL) interface—typically formed by fullerene (C_60_)—where severe nonradiative recombination and energy‐level mismatch occur [4]. While various interfacial passivation strategies have demonstrated significant improvements in the efficiency and stability of single‐junction perovskite solar cells [5], these approaches are less effective in tandem architectures. Even under identical passivation treatments, perovskite/silicon tandem devices degrade more rapidly than perovskite single‐junction counterparts [6], indicating the existence of degradation mechanisms unique to the tandem architecture [7].

A key distinction arises from the direction of light incidence and the resulting carrier transport geometry. In perovskite single‐junction solar cells, illumination occurs from the glass/ITO side, where the perovskite layer is supported by a high‐dielectric ITO substrate that promotes efficient charge separation. In contrast, perovskite/silicon tandems are illuminated through the C_60_ layer. The inherently low dielectric constant of C_60_ (≈4) provides weak electrostatic screening [8], allowing strong coulombic attraction between photogenerated carriers and thereby increasing the probability of bimolecular recombination near the ETL interface and shortening the carrier diffusion length. This limitation becomes particularly critical in monolithic perovskite/silicon tandems, where the perovskite top cell must be relatively thick (typically 700–900 nm) to achieve conformal coverage over the textured silicon surface beneath [9]. Under these circumstances, carriers generated near the C_60_ interface must traverse a long distance to reach the buried hole transport layer (HTL) [10], and a limited carrier diffusion length causes severe carrier accumulation within the bulk, thereby accelerating ion migration and compromising device stability [11]. Consequently, the low‐dielectric environment at the perovskite/C_60_ interface fundamentally constrains both efficiency and operational stability in perovskite/silicon tandems.

Most of the currently reported organic passivation layers and inorganic dielectrics—including alkali metal fluorides (LiF_X_ [12], MgF_X_ [13]) and oxides (AlO_X_ [14], SiO_X_ [15])—can neutralize interfacial traps through chemical bonding or field‐effect passivation, but they are unable to alleviate the dielectric mismatch at the perovskite/ETL interface. Their intrinsically low dielectric constants provide insufficient charge screening [16] and fail to suppress phonon‐assisted recombination [17], particularly under the high carrier densities typical of tandem operation. Therefore, it is crucial to design dielectric‐engineered interfacial contacts that simultaneously enable effective defect passivation, strong electrostatic screening, reduced carrier accumulation, and efficient carrier extraction, thereby ensuring long‐term operational stability in perovskite/silicon tandem solar cells.

In this work, a niobium oxide (NbO_X_) based high‐dielectric‐constant electron‐selective contact is employed to enhance the interfacial quality between perovskite and C_60_. The high dielectric environment suppresses carrier trapping by reducing the capture radius of interfacial defects, while chemical passivation lowers the interface defect density through Pb–O bond formation with undercoordinated Pb^2+^ or PbI_2_ at the perovskite surface. These two effects together extend the carrier diffusion length to from 1.32 to 2.64 µm. In addition, the strong electron selectivity of NbO_X_ further optimizes energy‐level alignment and electron transport at the C_60_ interface. Consequently, single‐junction perovskite solar cells with a 1.68 eV bandgap achieve a PCE of 22.4%, retaining 91% of their initial efficiency after 650 h of maximum power point (MPP) tracking (ISOS‐L‐1). The 1 cm^2^ monolithic perovskite/silicon tandem device reaches a PCE of 32.0%, among the highest reported for perovskite/TOPCon tandems, with unencapsulated devices retaining their initial efficiency after 200 h of MPP tracking under ISOS‐L‐1 testing. These results underscore the pivotal role of extended carrier diffusion in achieving both stable operation and high efficiency in tandem architectures.

## 2 Results and Discussion

**FIGURE 1:**
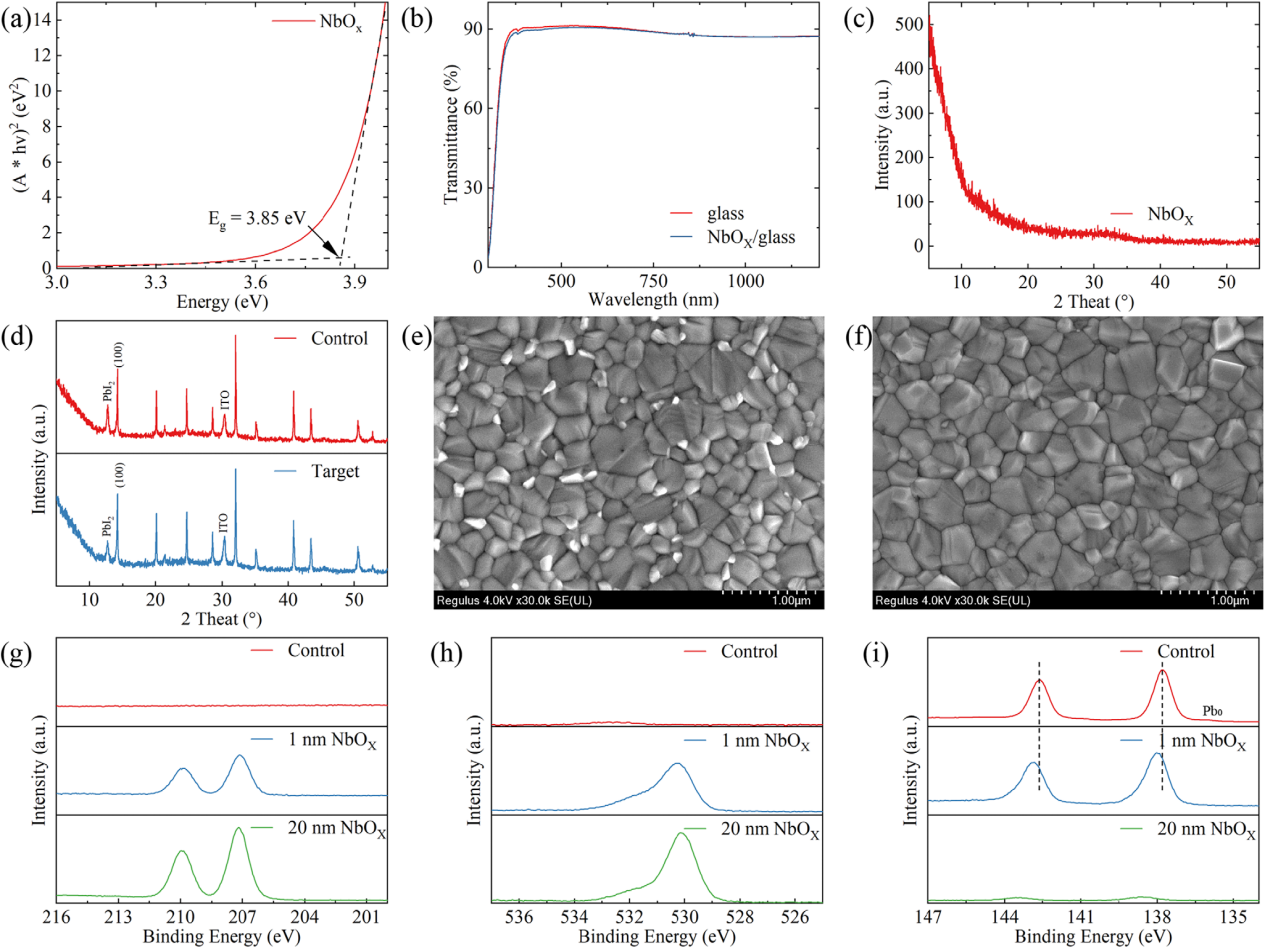
Optical and structural properties of NbO_X_ films and their effects and interactions with perovskite. a) Optical bandgap, b) transmittance spectra, and c) XRD patterns of NbO_X_ films. d) XRD patterns of perovskite films before and after NbO_X_ deposition. SEM images of perovskite films e) before and f) after NbO_X_ deposition. Magnification for all SEM images: ×30,000. XPS analysis of g) Nb 3d, h) O 1s, and i) Pb 4f on the perovskite surface after deposition of NbO_X_ with varying thicknesses.

An ultrathin NbO_X_ interlayer was deposited at the interface between the 1.68 eV wide‐bandgap triple‐cation mixed‐halide perovskite Cs_0.05_(FA_0.77_MA_0.23_)_0.95_Pb(I_0.77_Br_0.23_)_3_ absorber and the C_60_ ETL. UV–vis absorption spectra revealed that the deposited NbO_X_ film possesses an optical bandgap of approximately 3.4 eV (Figure 1a) and exhibits high optical transmittance comparable to that of the quartz substrate across the 300–1200 nm wavelength range (Figure 1b). Such a wide bandgap and excellent transparency ensure minimal optical loss within the passivation layer itself and preserve the light‐harvesting capability of the perovskite absorber in both the visible and near‐infrared regions. This optical property is particularly important for tandem devices, where the transmission of near‐infrared light to the underlying silicon subcell critically determines the overall spectral response and photocurrent balance. X‐ray diffraction (XRD) analysis further revealed the structural characteristics of the NbO_X_ film. No discernible diffraction peaks were observed, indicating that the film is amorphous (Figure 1c), consistent with previous reports on low‐temperature‐deposited NbO_X_ thin films [18]. The amorphous nature of NbO_X_ is advantageous for interfacial engineering, as the absence of grain boundaries mitigates stress concentration and prevents the formation of grain‐boundary traps [19]. Moreover, the smooth and uniform morphology facilitates intimate interfacial contact with the perovskite layer, minimizing pinholes and uncovered regions, thereby suppressing nonradiative recombination and promoting uniform electron transport.

After confirming the excellent optical transparency and amorphous nature of the NbO_X_ interlayer, we further investigated how its deposition affects the crystal structure of the perovskite absorber. X‐ray diffraction (XRD) patterns of the perovskite films with and without NbO_X_ are shown in Figure 1d. The diffraction peak corresponding to the (100) plane appears at 2θ = 14.2°, and the full width at half maximum (FWHM) as well as the relative intensity ratio of the PbI_2_ peak (2θ = 12.7°) to the (100) peak are summarized in Table S1. Compared with the control sample, the FWHM of the (100) peak slightly narrows from 0.135° to 0.122° after NbO_X_ deposition, indicating improved crystalline ordering. Meanwhile, the intensity ratio of PbI_2_ to (100) decreases significantly from 0.539 to 0.405, suggesting a reduced PbI_2_ content at the perovskite surface [20]. This reduction is likely attributed to interfacial reactions between oxygen species in the NbO_X_ film and the unsaturated Pb or PbI_2_ residues on the perovskite surface, forming Pb─O bonds that effectively passivate uncoordinated Pb^2+^ defect sites [15]. Moreover, the deposition of NbO_X_ does not alter the intrinsic perovskite crystal structure, as the positions and shapes of all characteristic diffraction peaks remain unchanged compared with the control sample. This confirms that the deposition process is nondestructive to the perovskite lattice [21]. In addition, no new diffraction peaks are observed after NbO_X_ deposition, consistent with the amorphous nature of the NbO_X_ film discussed above, further excluding the possibility of new phase formation or undesirable by‐products during the process. The surface morphology observed by scanning electron microscopy (SEM) further supports these findings. Although the perovskite grain size remains nearly unchanged (Figure S1), the bright PbI_2_‐enriched regions (Figure 1e) on the film surface are markedly reduced after NbO_X_ deposition (Figure 1f). This indicates that the conformal NbO_X_ coverage effectively suppresses the distribution of surface recombination sites, thereby facilitating a more uniform carrier extraction pathway at the perovskite/ETL interface.

To gain deeper insight into the interfacial interaction mechanism between NbO_X_ and the perovskite surface, X‐ray photoelectron spectroscopy (XPS) measurements were performed to examine the chemical composition changes of the perovskite films before and after NbO_X_ deposition with different thicknesses. As shown in Figure 1g, distinct Nb 3d peaks appear at binding energies of approximately 207.2 and 209.9 eV. The Nb 3d signal intensity increases with increasing NbO_X_ thickness, and the peak positions closely match those of stoichiometric Nb_2_O_5_, indicating that niobium in the deposited film exists predominantly in a high oxidation state. Concurrently, the O 1s peak intensity significantly increases after NbO_X_ deposition (Figure 1h), further confirming the presence of oxygen‐containing Nb compounds at the perovskite surface. Upon the deposition of 1 nm NbO_X_, a new oxygen species emerged in the O 1s spectrum compared to both the pristine perovskite and the pure NbO_X_ film (Figure S2). Deconvolution analysis identifies this new signal at ≈530.5 eV as the Pb─O bond, which is distinct from the lattice oxygen of NbO_X_ (Nb─O at ≈530.0 eV). The emergence of this specific coordination peak confirms that the NbO_X_ overlayer effectively passivates the perovskite surface defects by forming robust chemical bonds with under‐coordinated Pb^2+^ ions. As the thickness increases, the Nb─O signal strengthens progressively, reflecting the development of the oxide matrix. Notably, the persistence of a prominent Pb─O signal even at greater thicknesses demonstrates the formation of a stable and enduring interfacial passivation layer. This feature is corroborated by the Pb 4f spectrum, which shifts toward higher binding energy after NbO_X_ deposition (Figure 1i), implying chemical interactions between oxygen species and surface Pb^2+^ ions that lead to the formation of stable Pb─O bonds. The formation of Pb─O bond not only maintains efficient charge transport but also passivates interfacial defects [22], thereby reducing nonradiative recombination losses. The formation of Pb─O bonds was further corroborated by Fourier‐transform infrared (FTIR) spectroscopy (Figure S3), in which the target sample exhibited a distinctive new vibrational mode at approximately 780 cm^−^^1^, providing unambiguous evidence of the robust chemical coordination between the NbO_X_ overlayer and the perovskite surface. In the control sample, a characteristic metallic Pb^0^ peak appears at a binding energy of approximately 136.84 eV, which is typically associated with deep‐level trap states that severely deteriorate photovoltaic performance. After depositing a 1 nm NbO_X_ layer, the Pb^0^ signal is markedly suppressed, indicating that NbO_X_ effectively inhibits the generation of metallic lead defects and thus reduces the density of deep‐level recombination centers. This observation is consistent with the XRD results, which revealed a reduction in PbI_2_ content and the formation of Pb–O bonds, jointly confirming the dual role of NbO_X_ in chemical passivation and suppression of deep‐level defects. When the NbO_X_ thickness is further increased to 20 nm, the Pb signal intensity becomes significantly weaker, indicating that the perovskite surface is fully covered by the NbO_X_ overlayer.

**FIGURE 2:**
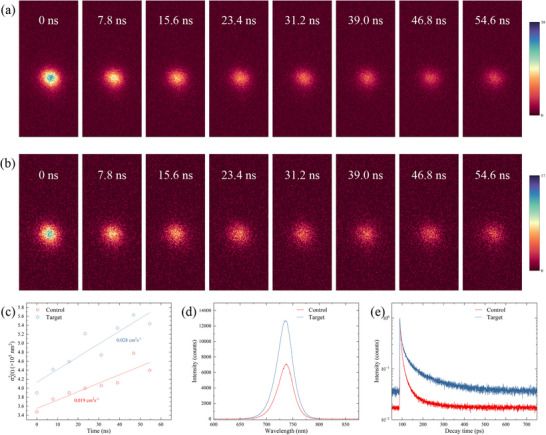
Effect of NbO_X_ on the carrier dynamics of perovskite films. PL intensity images of perovskite films at different excitation times a) before and b) after NbO_X_ deposition, where the lateral expansion of the PL spot over time reflects carrier transport from the excitation position to surrounding regions. c) The σ^2^_X_(t) vs. excitation time trend, from which the carrier diffusion coefficient can be obtained by linear fitting. d) Steady‐state PL spectra and e) time‐resolved PL spectra of perovskite films.

To investigate the effect of the high‐dielectric‐constant NbO_X_ layer on the carrier diffusion length of perovskite films, laser‐scanning time‐resolved fluorescence lifetime imaging microscopy (FLIM) was employed to visualize the spatiotemporal evolution of carrier transport [23]. A focused laser pulse locally excites carriers in a fixed region of the film, and photoluminescence (PL) images collected at successive delay times trace the diffusion of carriers away from the excitation center (Figure 2a,b). With increasing delay, the PL spot expands laterally, reflecting outward carrier diffusion. Gaussian fitting of the PL profiles (Figure S4) at different delay times and linear regression of the variance (Figure 2c) yield diffusion coefficients of 0.019 cm^2^ s^−^^1^ for the control and 0.028 cm^2^ s^−^^1^ for the NbO_X_‐passivated film. Beyond the diffusion coefficient, the carrier diffusion length is also governed by the carrier lifetime. We therefore examined the influence of NbO_X_ on carrier recombination dynamics. The absorption spectra of the perovskite films with and without NbO_X_ are nearly identical in the visible range, indicating that the NbO_X_ layer introduces negligible optical loss (Figure S5). In contrast, the steady‐state PL (SSPL) spectra (Figure 2d) show a pronounced enhancement in emission intensity and a narrower full width at half maximum (FWHM) after NbO_X_ deposition, signifying substantial suppression of nonradiative recombination at the perovskite surface. Time‐resolved PL (TRPL) measurements (Figure 2e) further quantify this effect. The decay curves, fitted with a bi‐exponential model (Table S2), reveal that for the control film the fast and slow decay lifetimes (τ_1_ and τ_2_) are 5.349 and 38.589 ns, respectively, whereas the NbO_X_‐passivated film exhibits a slightly longer τ_1_ of 9.017 ns and a markedly extended τ_2_ of 102.562 ns. These results demonstrate that NbO_X_ effectively passivates deep‐level traps at both the surface and interface, reducing nonradiative recombination. The effective carrier lifetime (τ_eff_) increases by nearly threefold, consistent with the enhanced PL intensity observed in SSPL. The decay‐component analysis reveals an increased contribution from radiative recombination (A_2_) and a reduced fraction of nonradiative processes, including trap‐assisted and interfacial recombination (A_1_). Combining the measured diffusion coefficients with the TRPL‐derived carrier lifetimes, the corresponding diffusion length increases from 1.32 µm to approximately 2.64 µm after NbO_X_ deposition. This improvement can be attributed to two synergistic effects: (i) the high dielectric constant of NbO_X_ (Figure S6), which mitigates coulombic interactions and reduces the defect capture radius at the interface through dielectric screening, and (ii) the chemical passivation provided by NbO_X_, where the formation of Pb─O bonds eliminates deep‐level defects on the perovskite surface, thereby suppressing recombination and enhancing carrier diffusion. Similar results are further confirmed by the two‐dimensional lifetime (Figures S7 and S8) and PL (Figure S9) mapping results.

**FIGURE 3:**
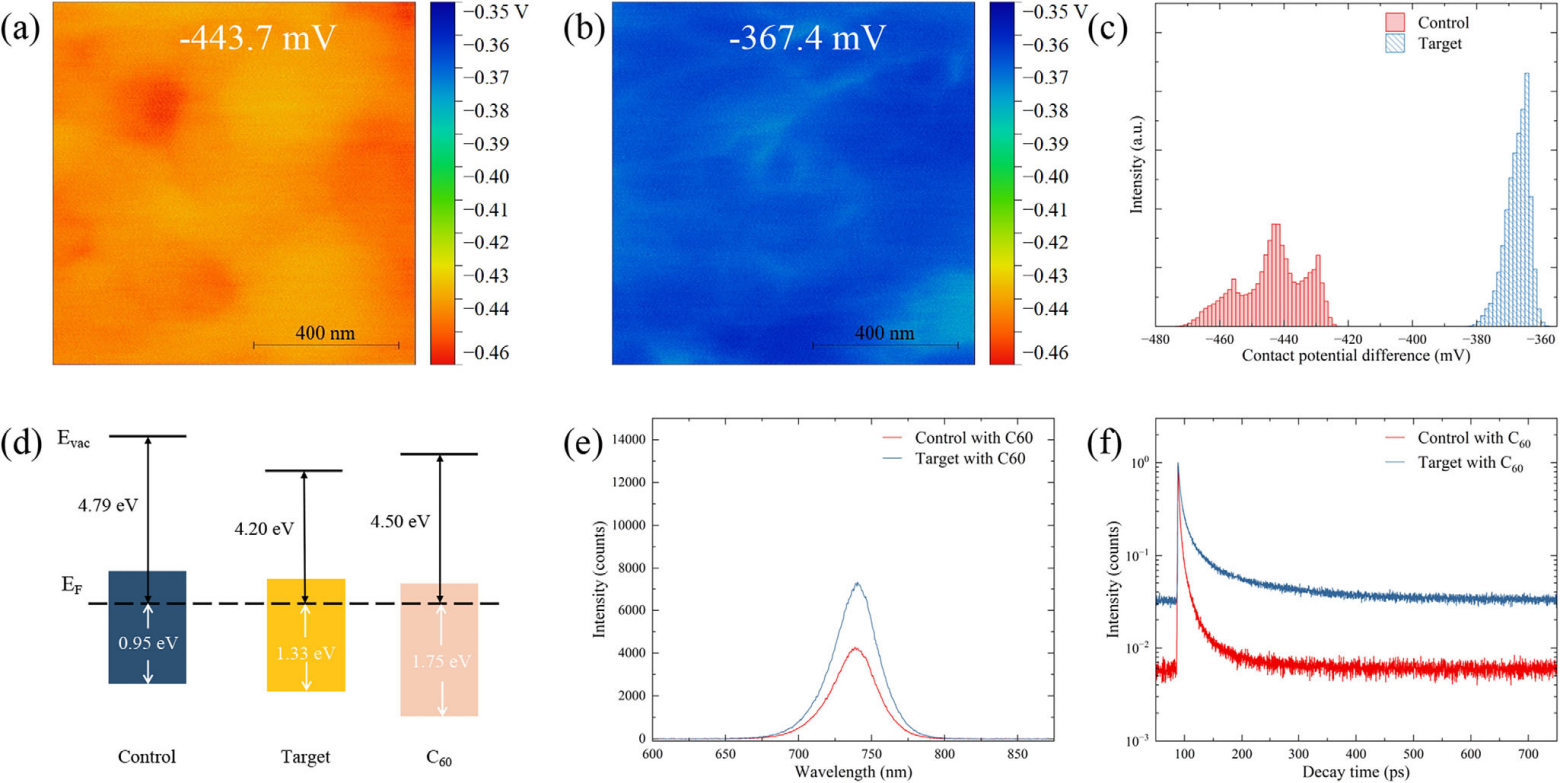
Effect of NbO_X_ on the perovskite/C_60_ contacts. Surface potential of perovskite a) before and b) after NbO_X_ deposition. Scale bars: 400 nm. c) Corresponding potential distribution map. d) Schematic of the energy‐level alignment between perovskite and C_60_. e) Steady‐state PL spectra and f) time‐resolved PL spectra of perovskite films with C_60_ capping layers.

To further elucidate the effect of the NbO_X_ layer on the energy‐level structure of the perovskite surface, scanning probe microscopy (SPM) measurements were performed in both atomic force microscopy (AFM) and Kelvin probe force microscopy (KPFM) modes to examine changes in surface potential distribution before and after NbO_X_ deposition. As shown in Figure S10, the surface morphology of the perovskite film remains essentially unchanged after NbO_X_ deposition, indicating that the conformal evaporation process does not affect the surface topography. The contact potential difference (CPD) distribution closely follows the surface morphology in both cases. The average CPD value of the pristine perovskite surface is −443.7 mV (Figure 3a), while that of the NbO_X_‐coated sample increases markedly to −367.4 mV (Figure 3b), accompanied by a more homogeneous potential distribution (Figure 3c). This shift in CPD suggests that NbO_X_ not only effectively passivates interfacial defects but also modulates the surface energy‐level alignment of the perovskite, leading to improved interfacial energetics. To quantitatively analyze these changes, ultraviolet photoelectron spectroscopy (UPS) was further employed to determine the electronic structure parameters of the perovskite surface. Consistent with the KPFM results, the work function of the perovskite decreases from 4.79 to 4.20 eV after NbO_X_ deposition (Figure S11). Based on the measured work function and optical bandgap, the valence‐band maximum (VBM) and conduction‐band minimum (CBM) positions were constructed to visualize the energy‐level alignment at the interface (Figure 3d) [24]. Compared with the control sample, the energy separation between the Fermi level and the CBM of the perovskite decreases by approximately 0.38 eV after NbO_X_ deposition, indicating an enhanced n‐type character of the perovskite surface. Moreover, the conduction‐band offset (CBO) between the perovskite and C_60_ is reduced from 0.57 to 0.37 eV, substantially mitigating interfacial electron accumulation and thereby suppressing nonradiative recombination and minimizing V_OC_ losses. In addition, NbO_X_ possesses a deep highest occupied molecular orbital (HOMO) level that effectively blocks hole backflow into the C_60_ layer and prevents interfacial electron–hole recombination. Its intrinsically high dielectric constant further contributes to dielectric screening, which shortens the capture radius of interfacial traps for free carriers. This dual effect facilitates rapid and directional electron transport toward the ETL, thereby enabling more efficient and selective charge extraction across the perovskite/ETL interface.

To probe how NbO_X_ influences carrier dynamics at the perovskite/C_60_ interface, steady‐state PL measurements were carried out after C_60_ deposition. As shown in Figure 3e, the PL intensity drops sharply for both samples, confirming efficient electron extraction by the C_60_ layer. Notably, the NbO_X_‐treated sample exhibits a higher residual PL intensity than the control. Such an increase could originate from two possible sources [25]: (i) improved interfacial quality that reduces nonradiative recombination at the perovskite/C_60_ contact, or (ii) a slight hindrance of electron transfer that increases radiative recombination. Therefore, time‐resolved PL analysis is further performed to disentangle these effects. As shown in Figure 3f and Table S3, the fast decay lifetime (τ_1_) slightly increases after NbO_X_ deposition, indicating that the impact on interfacial carrier transfer is minimal. In contrast, the slow decay component (τ_2_) extends from 22.8 to 94.3 ns, reflecting suppression of deep‐trap‐mediated recombination. Consequently, the effective lifetime (τ_eff_) increases from 0.4 to 1.7 µs, confirming the increased carrier diffusion length enabled by NbO_X_. These results demonstrate that NbO_X_ does not hinder electron injection into C_60_ but instead passivates interfacial traps and mitigates nonradiative recombination, thereby improving overall optoelectronic quality. Spatially resolved lifetime imaging (Figure S12) provides further evidence of the improved interfacial quality. In the control sample, τ_2_ decreases to ≈10 ns after C_60_ deposition, indicating the presence of numerous interfacial nonradiative centers that severely limit carrier lifetime. Upon introducing the NbO_X_ interlayer, τ_2_ increases significantly to ≈70 ns, confirming effective suppression of interfacial defects. Corresponding lifetime‐weighted maps (Figure S13) display higher average lifetimes and markedly more homogeneous spatial distributions, with low‐lifetime regions largely eliminated. These results demonstrate that NbO_X_ effectively reduces interfacial trap density and enhances grain‐boundary passivation, leading to improved spatial uniformity and local stability—consistent with the TRPL trends discussed above. Two‐dimensional PL mapping provides further confirmation of the improved contact quality (Figure S14). The target film exhibits not only a slightly higher overall PL intensity but also a notably smoother and more continuous emission profile, indicative of enhanced spatial uniformity [26]. Moreover, the emission peak becomes more uniform, shifting from 748 ± 6 nm in the control to 747 ± 2 nm after passivation (Figure S15), accompanied by a narrowing of the FWHM from 45.4 to 43.3 nm (Figure S16). The convergence of emission energy levels further reflects reduced energetic disorder and improved charge‐transport characteristics. Collectively, these optical and dynamic analyses demonstrate that NbO_X_ passivation effectively mitigates both surface and interfacial defects, extends carrier lifetime, and enhances electron‐transfer uniformity at the perovskite/C_60_ junction.

**FIGURE 4:**
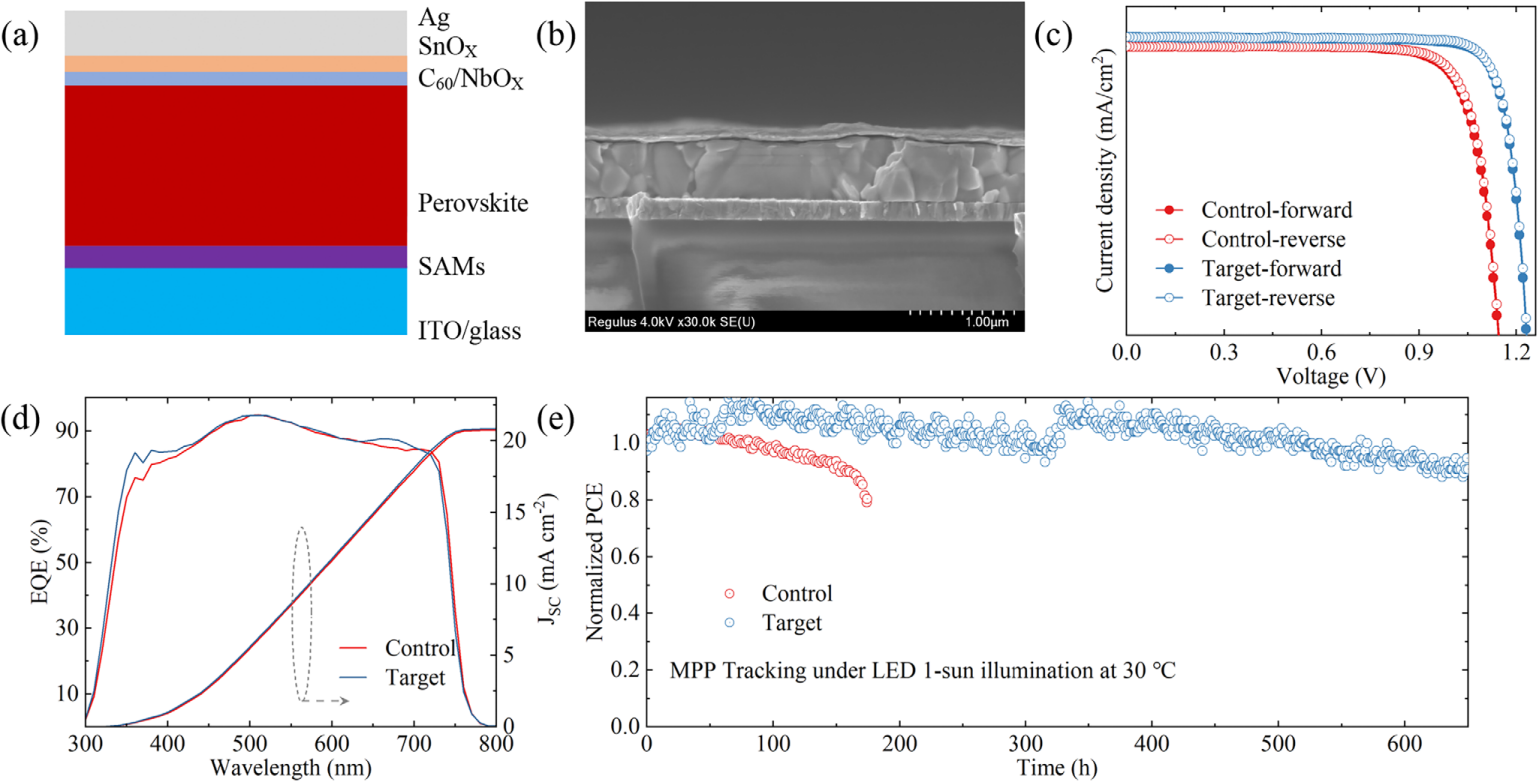
Effect of NbO_X_ on the performance of perovskite single‐junction devices. a) Schematic of the device structure, b) corresponding cross‐sectional SEM image, c) *J–V* curves, d) EQE spectra and corresponding integrated J_SC_, and e) operational stability of perovskite single‐junction solar cells with and without NbO_X_ passivation.

In order to evaluate the effect of NbO_X_ on the photovoltaic performance of wide bandgap perovskite solar cells, inverted devices with a structure of glass/ITO/4PADCB/Perovskite/NbO_X_/C_60_/SnO_X_/IZO/Ag were fabricated (Figure 4a). Cross‐sectional SEM confirms a layer stack consistent with the designed architecture (Figure 4b). Device performance was evaluated with varying NbO_X_ thicknesses to identify the optimal interlayer thickness (Table S4). The *J–V* characteristics of the champion devices are presented in Figure 4c, with the corresponding photovoltaic parameters summarized in Table S5. The control device exhibits a champion PCE of 19.3%, with V_OC_ = 1.15 V, J_SC_ = 21.1 mA cm^−2^, and FF = 79.9%. Upon introducing the NbO_X_ interlayer, the optimized device achieves an enhanced PCE of 22.4%, with V_OC_ = 1.23 V, J_SC_ = 21.7 mA cm^−2^, and FF = 83.4%. In addition to the obvious increase in PCE, the NbO_X_‐modified device shows reduced hysteresis compared with the control, indicating improved carrier extraction and mitigated charge accumulation. The external quantum efficiency (EQE) spectrum of the NbO_X_ device (Figure 4d) yields an integrated J_SC_ of 20.9 mA cm^−2^, consistent with the value obtained from the *J–V* measurements. Under continuous one‐sun illumination at the maximum power point, the target device maintains a stabilized power output (SPO) of 22.3% after 300 s (Figure S17), confirming its excellent operational stability. To assess the reproducibility of the NbO_X_ modification, 20 control and target devices were fabricated within the same batch. The statistical photovoltaic parameters (Figure S18) reveal that the target devices exhibit consistently higher performance and narrower parameter distributions, demonstrating improved reproducibility. The enhanced PCE of the target device primarily arises from the simultaneous improvements in V_OC_ and FF. The high dielectric constant and chemical passivation capability of NbO_X_ effectively suppress interfacial defects and reduce nonradiative recombination, leading to an increased V_OC_. Meanwhile, the favorable energy‐level alignment at the perovskite/C_60_ interface facilitates efficient electron extraction and minimizes interfacial barriers, thereby enhancing FF. Together, these effects confirm that NbO_X_ serves as an efficient interfacial passivation and charge transport layer, substantially improving both the efficiency and reproducibility of devices.

The impact of NbO_X_ on the stability of perovskite films and devices was further investigated. Both control and NbO_X_‐modified perovskite films were annealed at 85°C for various durations, and their phase evolution was systematically monitored. After 200 h, the control film exhibited a pronounced growth of the PbI_2_ diffraction peak at 2θ = 12.7°, indicative of perovskite decomposition (Figure S19a). In contrast, the target film retained a dominant perovskite (100) peak near 14.1°, with negligible PbI_2_ formation, demonstrating superior structural robustness (Figure S19b). Furthermore, the operational stability of the corresponding devices was evaluated through maximum power point (MPP) tracking under continuous AM 1.5 G illumination without a UV cutoff filter in ambient conditions. As shown in Figure 4e, the NbO_X_‐based device exhibits significantly enhanced MPP stability, retaining over 91% of its initial PCE after 650 h, whereas the control device degraded to 80% after 170 h. To further assess the long‐term operational potential, we subjected the devices to protracted thermal stress at 85°C (Figure S20). Linear extrapolation of the degradation trajectory indicates that the target device can maintain over 85% of its initial efficiency for more than 1000 h. While the control device underwent rapid performance decay, dropping to 70% of its original PCE within 90 h, the NbO_X_‐passivated device showcased exceptional durability, preserving 90% of its efficiency beyond 270 h. This remarkable improvement in thermal and operational stability is attributed to the extended carrier diffusion length, effective suppression of surface defects, and the formation of strong interfacial bonding. The robust NbO_X_ interlayer also acts as a protective barrier, mitigating organic cation volatilization and preserving the structural integrity of the perovskite absorber during prolonged operation.

To elucidate the mechanism behind the performance gains, we quantified the electronic trap density via the space‐charge‐limited current (SCLC) method (Figure S21) using ITO/SnO_X_/Perovskite/C_60_/Ag electron‐only devices. From the trap‐filled limit voltage VTFL, the trap density decreases from 3.73 × 10^15^ cm^−3^ (control) to 2.92 × 10^15^ cm^−3^ with NbO_X_, indicating effective suppression of interfacial electronic defects [27]. V_TFL_ also drops from 0.23 to 0.18 V, consistent with improved interfacial energetics and transport. Dark *J–V* characteristics of full devices (Figure S22) show reduced reverse‐bias leakage and higher forward‐bias injection for the NbO_X_ device, attributable to a more strongly n‐type perovskite surface after NbO_X_ deposition, which facilitates electron injection while lowering recombination [28]. We further probed the built‐in potential (V_bi_) by capacitance–voltage analysis using the Mott–Schottky relation (Figure S23):

$$\frac{1}{C^{2}}=\frac{2\left(V_{bi}-V\right)}{A^{2}e\varepsilon_{0}\varepsilon_{r}N_{A}}$$

where A is the active area, ε_0_ the vacuum permittivity, ε_r_ the relative permittivity, and N_A_ the carrier density [29]. V_bi_ increases from 0.95 V (control) to 1.08 V with NbO_X_, consistent with the higher V_OC_. A larger V_bi_ strengthens the internal field under operation, promoting carrier separation/transport and reducing interfacial recombination. Under open‐circuit, transient photovoltage decay (Figure S24) shows an extended carrier lifetime for the NbO_X_ device, indicating suppression of nonradiative pathways [30]. These results align with the SCLC and impedance analyses and reinforce that NbO_X_ combines energy‐level modulation with defect passivation to improve interfacial electronics. We also assessed recombination mechanisms via light‐intensity‐dependent V_OC_ and J_SC_. From:

$$V_{OC}=\frac{nkT}{q}\;ln\left(P\right)+const$$

the slope of V_OC_ vs log(P) yields the ideality factor n (Figure S25) [31]. The NbO_X_ device exhibits a slope consistent with reduced trap‐assisted recombination relative to the control, indicative of improved interfacial quality. The dependence of photocurrent follows [32]:

$$J_{SC}\propto P^{\alpha }$$

where α reflects bimolecular recombination under operating conditions (Figure S26). The NbO_X_ device shows α closer to 1, evidencing lower recombination loss at high carrier densities and more efficient charge extraction. Electrochemical impedance spectroscopy further reveals an increased recombination resistance R_rec_ with NbO_X_ (Figure S27) [33], corroborating the suppression of nonradiative recombination and the improved carrier survival and collection under bias. Moreover, thermal admittance spectroscopy reveals a reduced effective trap density and a shift of trap states toward shallower energy levels upon introduction of the ultrathin NbO_X_ layer (Figure S28), indicating effective defect passivation at the perovskite/NbO_X_ interface.

**FIGURE 5:**
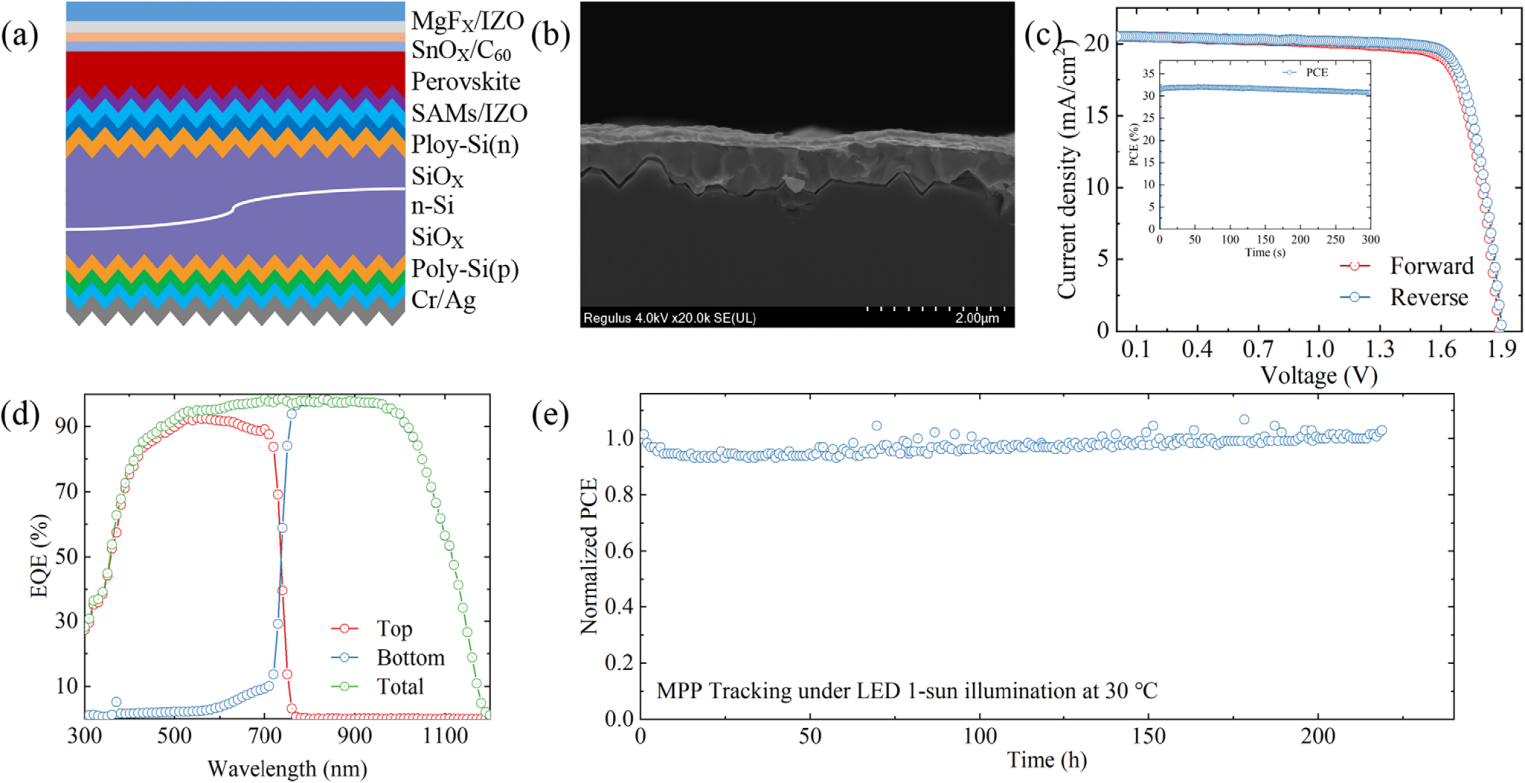
Device performance of perovskite/silicon tandems. a) Schematic of the tandem device structure, b) corresponding cross‐sectional SEM image, c) *J–V* curves, d) EQE spectra, and e) operational stability of perovskite/silicon tandem solar cells.

After verifying the performance enhancement in single‐junction devices, the NbO_X_‐passivated perovskite top cell was integrated onto a double‐sided textured TOPCon silicon bottom cell to fabricate a monolithic perovskite/silicon tandem solar cell (Figure 5a). To minimize optical reflection losses at the perovskite/silicon interface, the bottom cell surface employed submicron‐scale pyramidal texturing, enabling broadband photon trapping. For efficient charge recombination and high optical transmittance, the interconnecting recombination layer was constructed by combining a 1 nm‐thick 4PADCB self‐assembled monolayer with a 10 nm‐thick IZO film [34]. Cross‐sectional SEM imaging (Figure 5b) reveals that the perovskite film conformally and uniformly coats the submicron textured surface, with an average thickness of approximately 650 nm and vertically oriented grains spanning the entire film, indicative of high‐quality film formation. Benefiting from this optimized device architecture, the resulting tandem solar cell achieves an impressive power conversion efficiency (PCE) of 32.0%, with V_OC_ = 1.93 V, J_SC_ = 20.5 mA cm^−2^, and FF = 80.6% (Figure 5c; Table S6). The SPO test under AM 1.5 G illumination demonstrates a stabilized efficiency of 31.7% after 5 min of operation at the MPP, as shown in the inset of Figure 5c. The EQE spectra (Figure 5d) confirm well‐matched photocurrent generation between the subcells, with integrated current densities of 20.5 mA cm^−2^ for the perovskite top cell and 20.3 mA cm^−2^ for the silicon bottom cell, consistent with the *J–V* measurements. To assess the long‐term operational stability imparted by NbO_X_ passivation, unencapsulated tandem devices were subjected to continuous MPP tracking under ambient conditions (<40% RH, room temperature). The control device exhibited significant degradation, retaining only ∼80% of its initial PCE after 200 h of operation (Figure S29). In contrast, the NbO_X_‐passivated device maintained nearly constant output power over the same period (Figure 5e), demonstrating excellent operational stability.

## 3 Conclusion

In summary, we developed a dielectric‐engineered contact strategy employing a high‐dielectric NbO_X_ interlayer at the perovskite/C_60_ interface to simultaneously enhance the efficiency and long‐term operational stability of perovskite/silicon tandem solar cells. The high‐dielectric environment effectively suppresses defect‐mediated carrier trapping by reducing the capture radius of interfacial defects, while chemical passivation through Pb─O bond formation lowers the interface defect density. Together, these effects markedly extend the carrier diffusion length and mitigate carrier accumulation within the thick perovskite layer. Consequently, single‐junction perovskite solar cells with a 1.68 eV bandgap achieve a PCE of 22.4%, retaining 91% of their initial efficiency after 650 h of MPP tracking. Furthermore, 1 cm^2^ monolithic perovskite/silicon tandem devices reach a PCE of 32.0%, with unencapsulated devices maintaining 100% of their initial performance after 200 h of MPP tracking, underscoring the pivotal role of dielectric modulation in achieving both high efficiency and operational stability in tandem architectures.

## Author Contributions

Z.Y., X.Y., and J.Y. contributed to the conceptualization, funding acquisition, and project administration. W.L., Z.Y., X.Y., and J.Y. were responsible for the methodology and investigation. W.L., Z.Y., X.Y., and J.Y. wrote the original draft, and W.L., Z.Y., X.Y., and J.Y. contributed to review and editing. J.Y. contributed to the supervision. W.L., Z.Y., H.L., H.M., Y.Y., R.L., M.Z., Y.Z., and L.Z. contributed to the visualization.

## Conflicts of Interest

The authors declare no conflicts of interest.

## Supporting information




**Supporting File**: advs74051‐sup‐0001‐SuppMat.docx.

## Data Availability

The data that support the findings of this study are available in the supplementary material of this article.
