# Supplementary material for: Extending Carrier Diffusion via Interfacial Dielectric Shielding for Operationally Stable Perovskite/TOPCon Tandem Solar Cells
Source: Adv Sci (Weinh). 2026 Jan 28;13(19):e24128. doi: 10.1002/advs.202524128 (PMC13045422; doi:10.1002/advs.202524128)
Supplement: Supplementary file 1 — Supporting File: advs74051‐sup‐0001‐SuppMat.docx. [file ADVS-13-e24128-s001.docx]

**Supplementary Materials for**

**Extending Carrier Diffusion via Interfacial Dielectric Shielding for Operationally Stable Perovskite/TOPCon Tandem Solar Cells**

Wenfeng Liu^1,3^, Zhiqin Ying^1^*, Huan Li^1^, Xin Li^1^, Haofan Ma^1^, Yunyun Yu^1^, Ziyu He^1^, Rui Li^1^, Meili Zhang^1^, Yuheng Zeng^1^, Luyao Zheng^1^, Jicheng Zhou^3^*, Xi Yang^1,2^* and Jichun Ye^1,2^*

^1^Ningbo Institute of Materials Technology and Engineering, Chinese Academy of Sciences (CAS), Ningbo, 315201, China

^2^Yongjiang Laboratory, Ningbo, Zhejiang, 315201, China

^3^School of Energy Science and Engineering, Central South University, Changsha, China

*Corresponding author: Zhiqin Ying; Jicheng Zhou; Xi Yang; Jichun Ye; Email: yingzhiqin@nimte.ac.cn; jicheng@csu.edu.cn; xi-yang@y-lab.ac.cn; jichun.ye@nimte.ac.cn

**This Supplementary Materials includes:**

Experimental Section

Supplementary Figs. 1 – 29,

Supplementary Tables 1 – 6,

**Experimental Section**

*Materials*: Pre-patterned indium tin oxide (ITO) glass substrates (sheet resistance: 7–9 Ω/sq, visible light transmittance ≥ 89%) were supplied by Advanced Election Technology Co., Ltd. Most solvents, including anhydrous ethanol (99.5%), N,N-dimethylformamide (DMF, 99.8%), dimethyl sulfoxide (DMSO, anhydrous), and chlorobenzene (CB, anhydrous, 99.5%), were purchased from Aladdin. Cesium iodide (CsI, 99.999%) was obtained from Sigma-Aldrich. Methylammonium bromide (MABr) and formamidinium iodide (FAI) were sourced from GreatCell Solar Ltd. (4-(7H-Dibenzo[c,g]carbazol-7-yl)butyl)phosphonic acid (4PADCB), lead(II) bromide (PbBr_2_), and lead(II) iodide (PbI_2_) were procured from TCI. C_60_ (99.5%) and bathocuproine (BCP, 99.5%) were provided by Lumtec Corp. Silver (Ag) particles and niobium(V) oxide (Nb_2_O_5_) particles were acquired from Fuzhou Invention Photoelectrical Technology. All chemicals were used as received without further purification.

*Fabrication of Single-junction Perovskite Solar Cells*: ITO glass substrates were sequentially ultrasonicated in deionized water, acetone, and ethanol for 10 min each, followed by UV-ozone treatment for 15 min. Inside a nitrogen-filled glovebox, a hole transport layer was deposited by spin-coating a 0.3 mg/mL solution of 4PADCB in ethanol at 4000 rpm for 30 s, followed by annealing at 100 °C for 10 min. The substrates were then dynamically rinsed by ethanol. Subsequently, the perovskite precursor solution was spin-coated at 3500 rpm for 40 s. At 20 s before the end of the process, 300 µL of CB anti-solvent was dripped onto the film. The resulting perovskite film was annealed at 100 °C for 20 min. A 2 nm-thick NbO_X_ layer was deposited via electron beam evaporation at a rate of 0.01 Å/s. Then, 20 nm of C_60_ was thermally evaporated at a rate of 0.2 Å/s, followed by the deposition of a15 nm SnO_X_ layer using atomic layer deposition. Finally, a 100 nm-thick Ag was fabricated using thermally evaporated at room temperature.

*Fabrication of Perovskite/Silicon Tandem Solar Cells*: The TOPCon silicon bottom cell was fabricated according to the procedure described in our previous reports. For the perovskite top cell, the cleaned substrates were ultrasonicated in ethanol for 3 min, followed by UV-ozone treatment for 15 min. Then the samples were transferred into a nitrogen glovebox (< 10 ppm O_2_). The deposition processes for the 4PADCB, perovskite, NbO_X_, C_60_, and SnO_X_ layers were identical to those used for the single-junction devices. A 80 nm-thick IZO electrode (sheet resistance: 44 Ω/sq) was then sputtered at room temperature using an RF power of 80 W. To minimize sputter-induced damage, the initial 10 nm of IZO was deposited at a reduced RF power of 30 W. Finally, top Ag grid electrodes (200 nm) were thermally evaporated through a shadow mask at a rate of 1.0 Å/s.

*Film Characterization*: The surface and cross-sectional morphologies of perovskite films and tandem devices were characterized using a Hitachi S-4800 field-emission gun scanning electron microscope (FEG-SEM). X-ray diffraction (XRD) patterns were acquired on a Bruker D8 ADVANCE DAVINCI diffractometer with Cu Kα radiation (λ = 1.5418 Å). UV–vis absorption spectra were recorded on a Perkin-Elmer Lambda 950 spectrophotometer. Steady-state photoluminescence (SSPL) and time-resolved photoluminescence (TRPL) spectra were collected using a HORIBA FL3-111 spectrometer with 450 nm excitation. Surface potential mapping was performed on a Dimension 3100 or Dimension ICON (Bruker) scanning probe microscope (SPM). X-ray photoelectron spectroscopy (XPS) and ultraviolet photoelectron spectroscopy (UPS) measurements were conducted on a Kratos Axis Ultra DLD system under ultrahigh vacuum (3.0 × 10^-8^ Torr), using a non-monochromatic He-I source (21.22 eV) for UPS.

*Device Characterization*: Current density–voltage (J–V) characteristics, dark J–V behavior for space-charge-limited current (SCLC) analysis, and open-circuit voltage (V_OC_) versus light intensity measurements were performed using an Enlitech SS-F5-3A solar simulator under standard AM 1.5G illumination (100 mW/cm^2^). J–V curves of single-junction perovskite solar cells were measured in ambient air through an aperture mask (active area: 0.1 cm^2^). External quantum efficiency (EQE) spectra were obtained using an EnliTech QE-R system, with light intensity calibrated using certified Si and Ge reference cells. For tandem device EQE measurement, the perovskite sub-cell was characterized while biasing the silicon sub-cell with >800 nm light, and the silicon sub-cell was measured under <800 nm bias light illumination, with applied bias voltages of ~0.5 V and ~1.0 V, respectively. All EQE tests were conducted in air without encapsulation. Electrochemical impedance spectroscopy (EIS), capacitance–frequency (C–F), and Mott–Schottky measurements were carried out using a CHI660E electrochemical workstation. Mott–Schottky analysis was performed in impedance–potential mode with a bias range from –1.2 V to 0.5 V. Transient photocurrent (TPC) and transient photovoltage (TPV) were measured using a custom-built system coupled with the CHI660E workstation. Operational stability testing for the monolithic perovskite/silicon tandem solar cell was performed at the maximum power point under simulated AM 1.5G spectrum (100 mW/cm^2^, xenon lamp, unfiltered). The unencapsulated device was tested in ambient air (relative humidity: 40–85%, typically ~80%; temperature: 25–35 °C) without any cooling throughout the measurement.


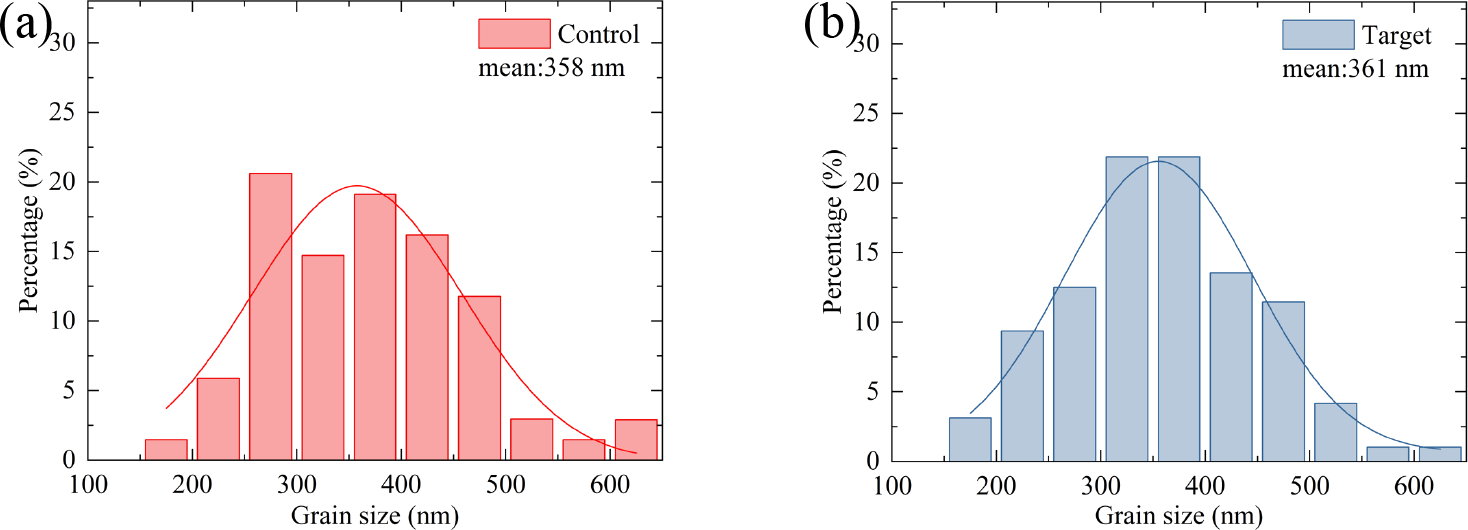


Supplementary Figure 1.Grain-size distribution histograms for a) control and b) target perovskite films


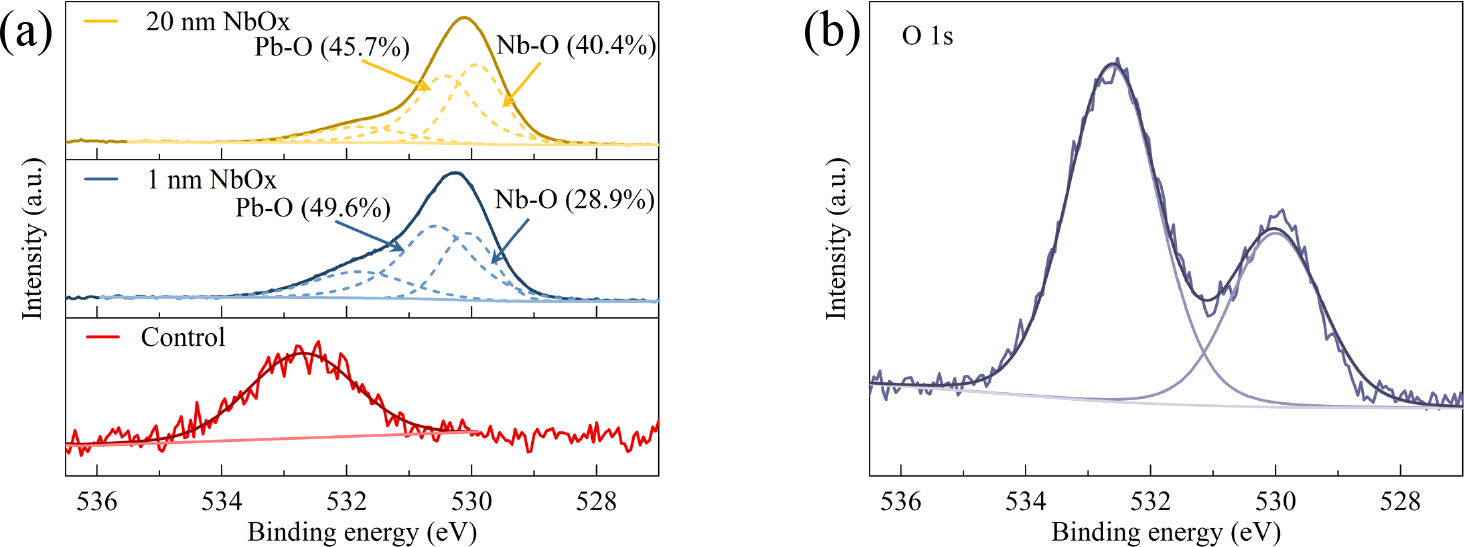


Supplementary Figure 2. a) High-resolution O 1s XPS spectra of the perovskite surface after deposition of NbO_X_ with different thicknesses; b) O 1s XPS spectrum of the pristine NbO_X_.


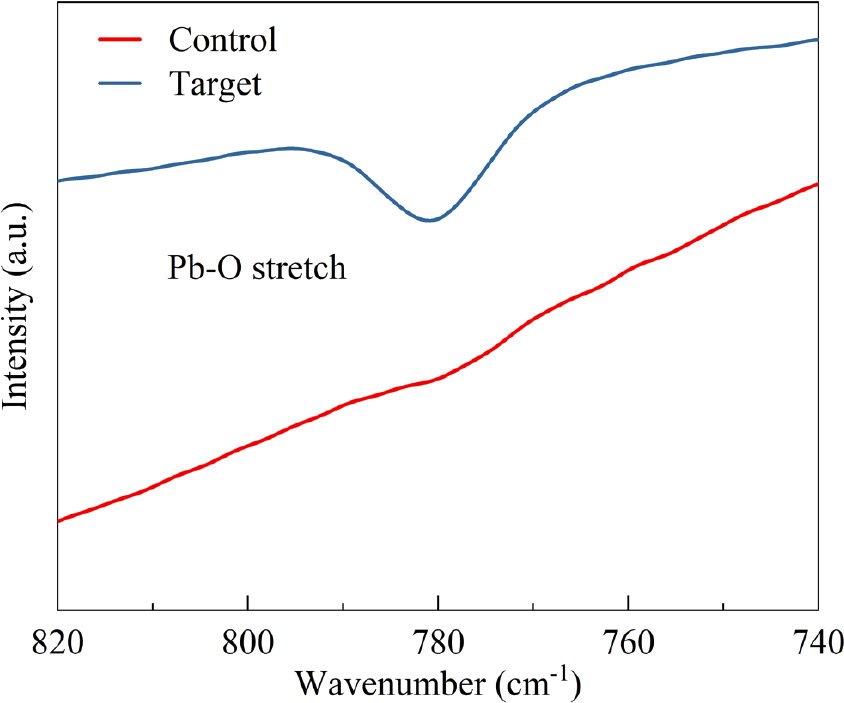


Supplementary Figure 3. FTIR spectra for perovskite films without and with the treatment of NbOX.


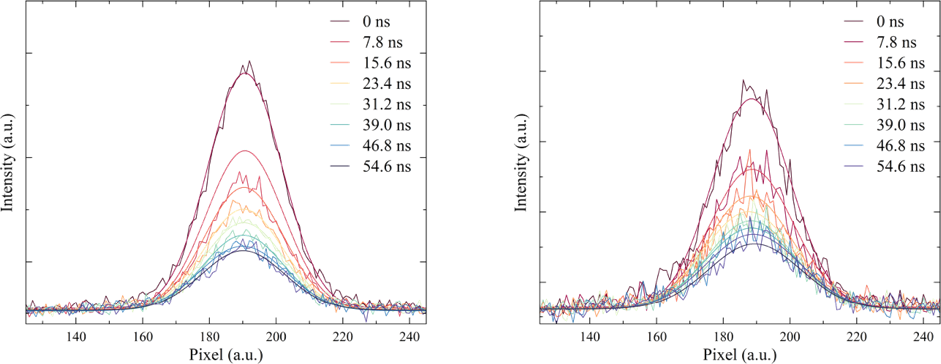


Supplementary Figure 4. Effect of NbO_X_ on the carrier diffusion length of perovskite films. Cross-sectional profiles of PL spots at different excitation times a) without and b) with NbO_X_.


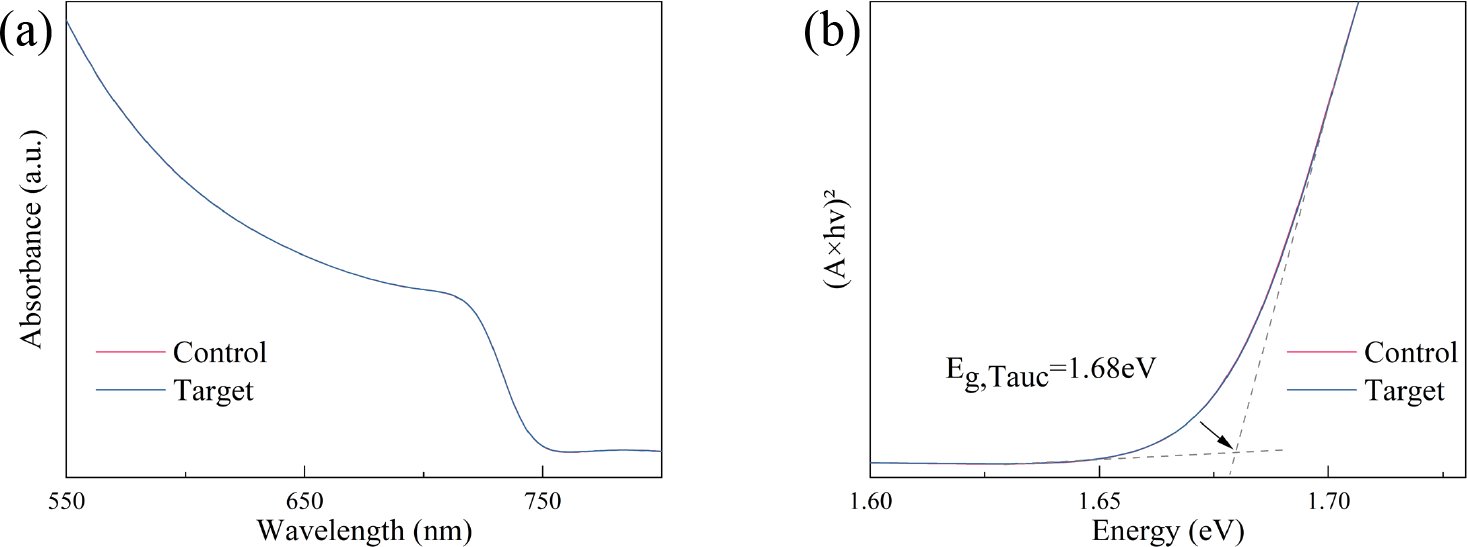


Supplementary Figure 5. UV–Vis absorption spectra and corresponding Tauc plots of the control and NbO_X_-treated perovskite films.


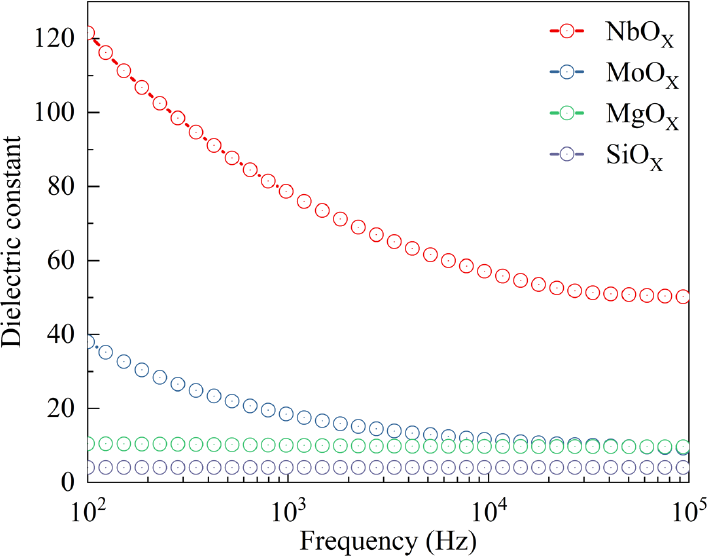


Supplementary Figure 6. Frequency-Dependent Dielectric Constant (εᵣ) of Various Metal Oxides


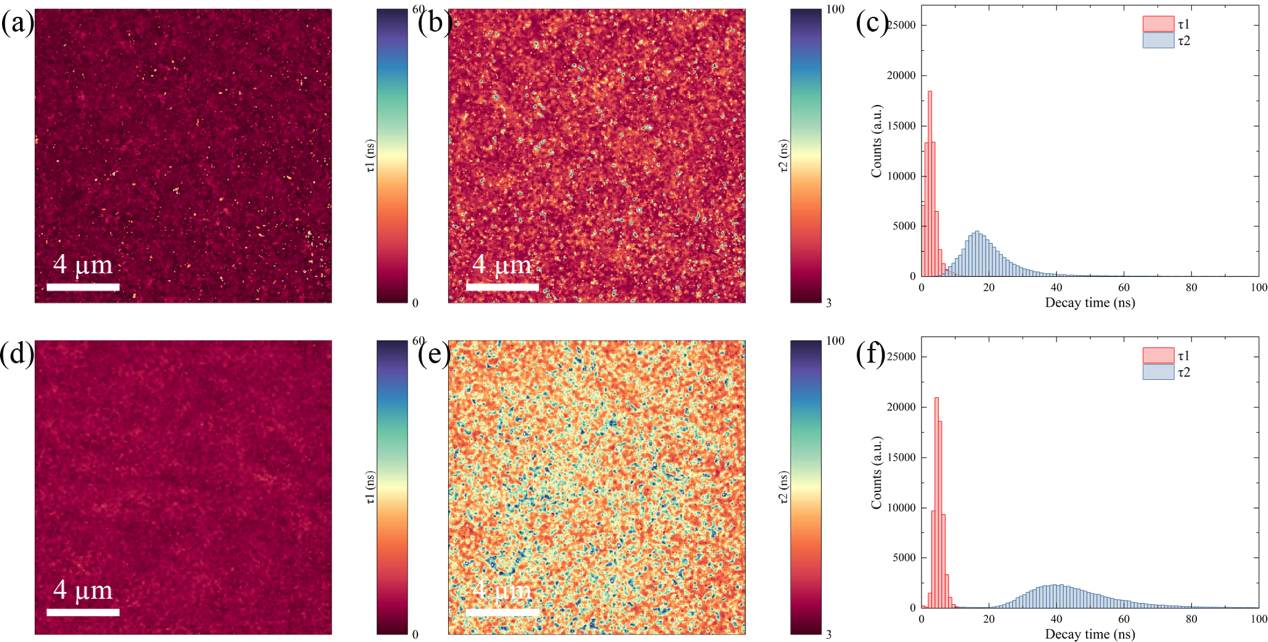


Supplementary Figure 7. Effect of NbO_X_ on carrier lifetimes of the perovskite. a) Fast lifetime, b) slow lifetime images, and c) corresponding lifetime distribution histogram of perovskite films before NbO_X_ deposition. d) Fast lifetime, e) slow lifetime images, and f) corresponding lifetime histogram of perovskite films after NbO_X_ deposition. Scale bars, 4 µm.


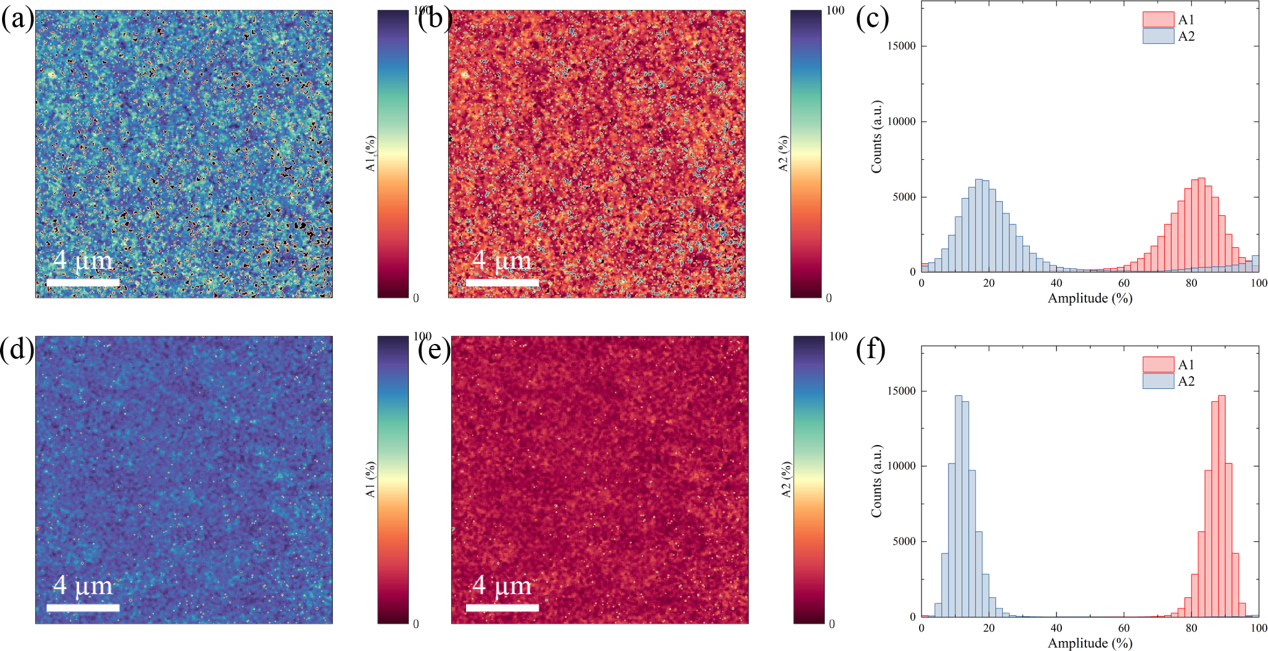


Supplementary Figure 8. Effect of NbO_X_ on the weights of fast and slow decay components of carriers at the perovskite surface. a) Fast lifetime weight, b) slow lifetime weight images, and c) corresponding lifetime weight distribution histogram for perovskite films before NbO_X_ deposition. d) Fast lifetime weight, e) slow lifetime weight images, and f) corresponding lifetime weight histogram for perovskite films after NbO_X_ deposition. Scale bars, 4 µm.


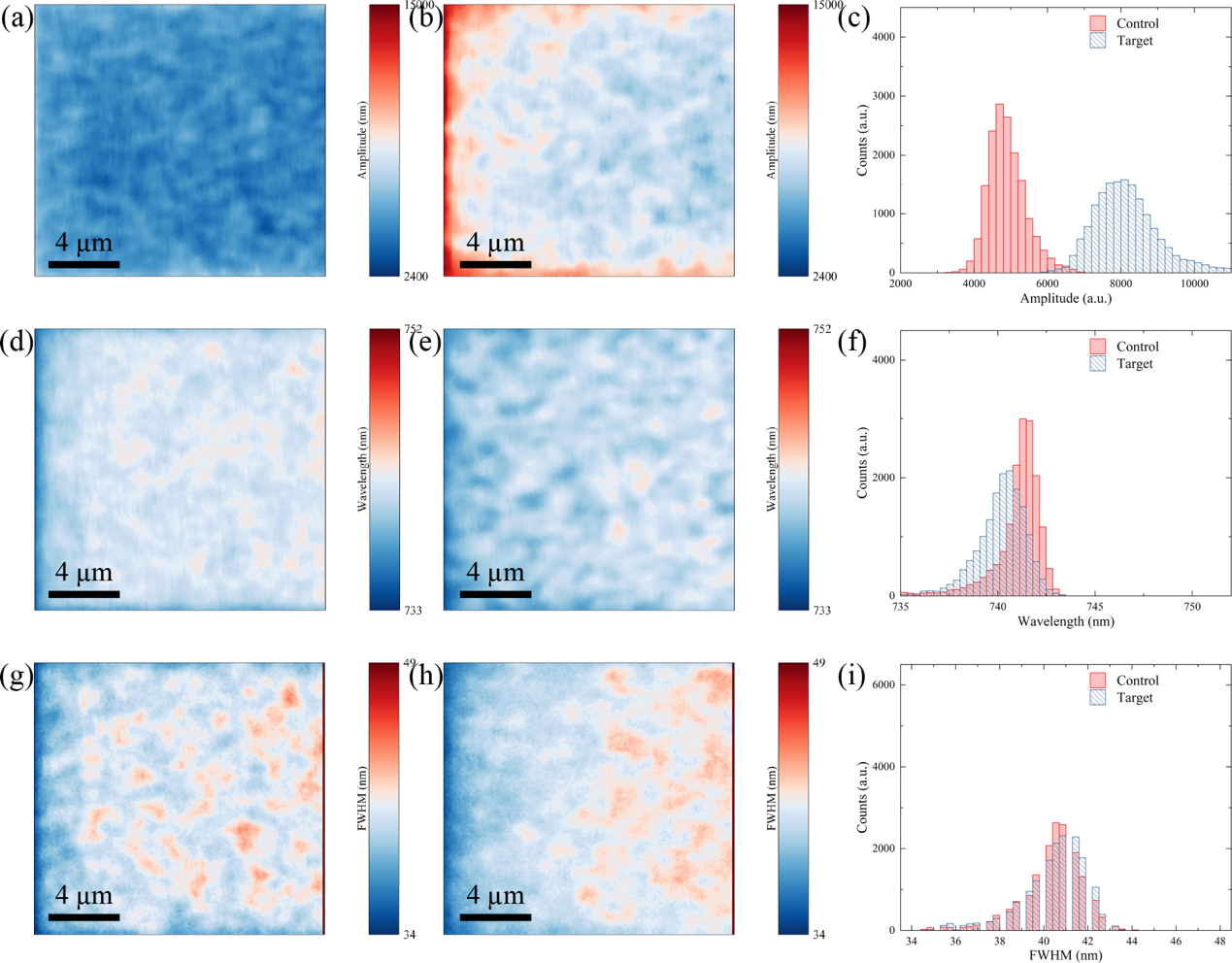


Supplementary Figure 9. Effect of NbO_X_ on the PL spectra of perovskite film surfaces. PL peak intensity images of perovskite films a) before and b) after NbO_X_ deposition, and c) corresponding PL peak intensity histogram; PL peak wavelength images d) before and e) after NbO_X_ deposition, and f) corresponding peak wavelength histogram; PL peak full width at half maximum images g) before and h) after NbO_X_ deposition, and i) corresponding PL peak FWHM histogram.


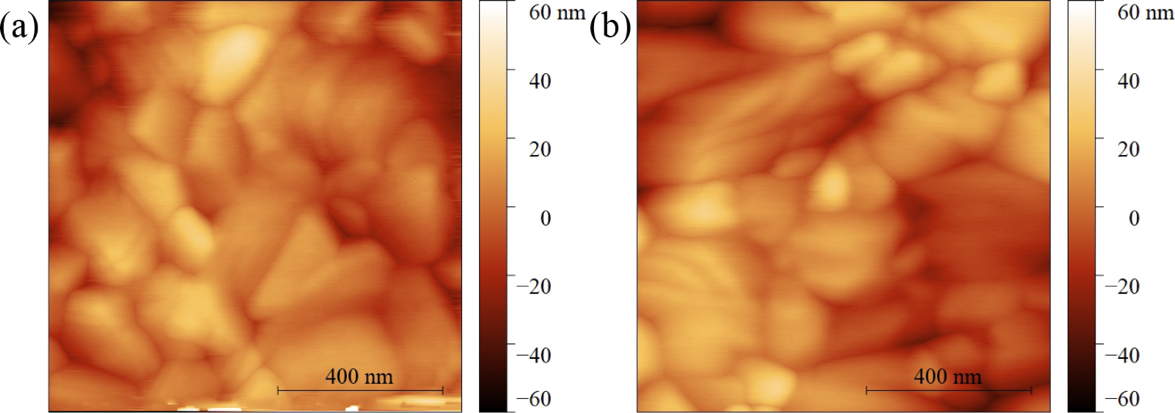


Supplementary Figure 10. Effect of NbO_X_ on the surface morphology of perovskite. Surface morphology of perovskite a) before and b) after NbO_X_ deposition. The root-mean-square (Rq) surface roughness decreases slightly from 18 nm to 15 nm after NbOₓ deposition.


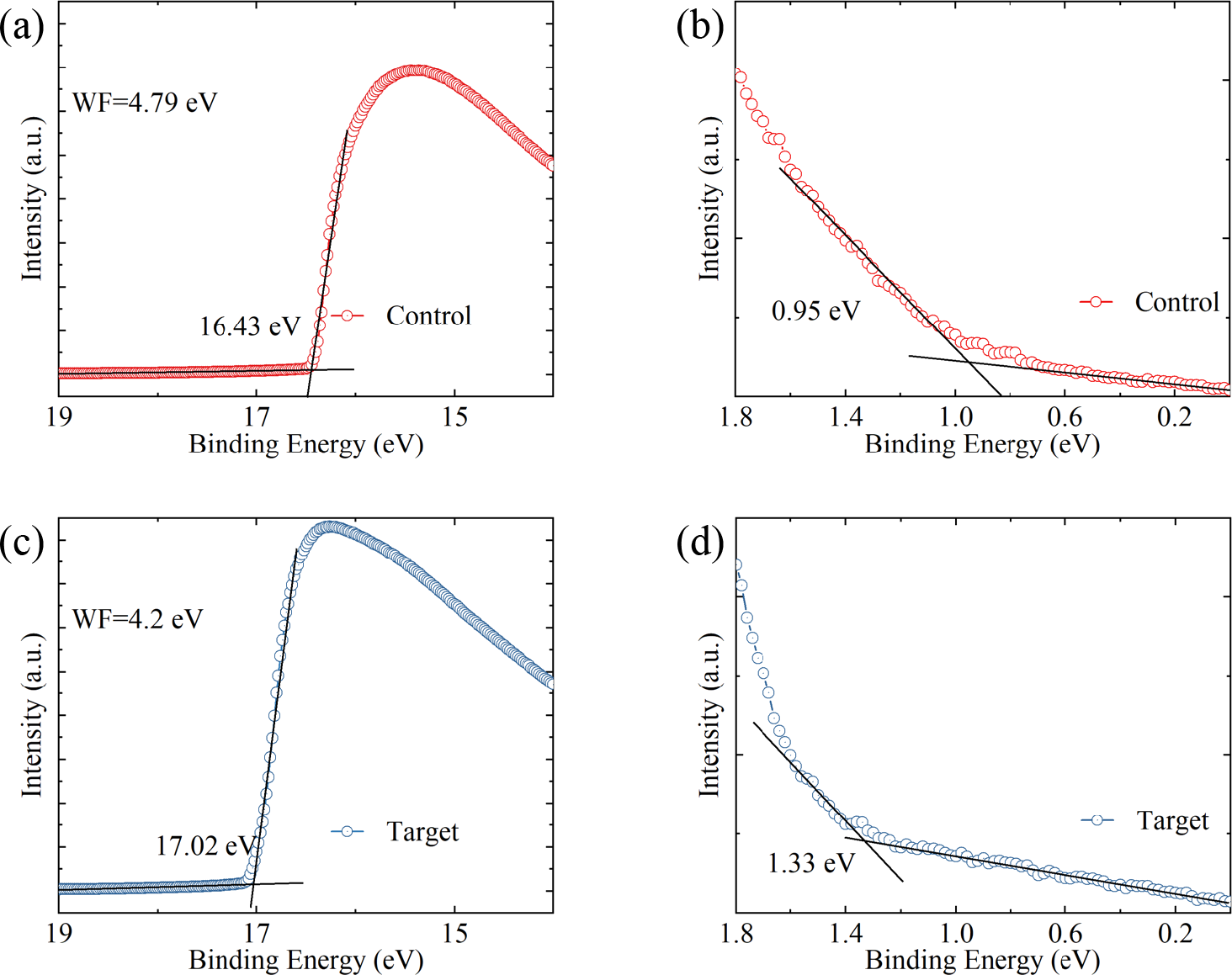


Supplementary Figure 11. Effect of NbO_X_ on the energy-level alignment of the perovskite surface. UPS spectra of the perovskite film a,b) before and c,d) after NbO_X_ deposition, showing the a,c) secondary electron cutoff and b,d) valence band edge regions.


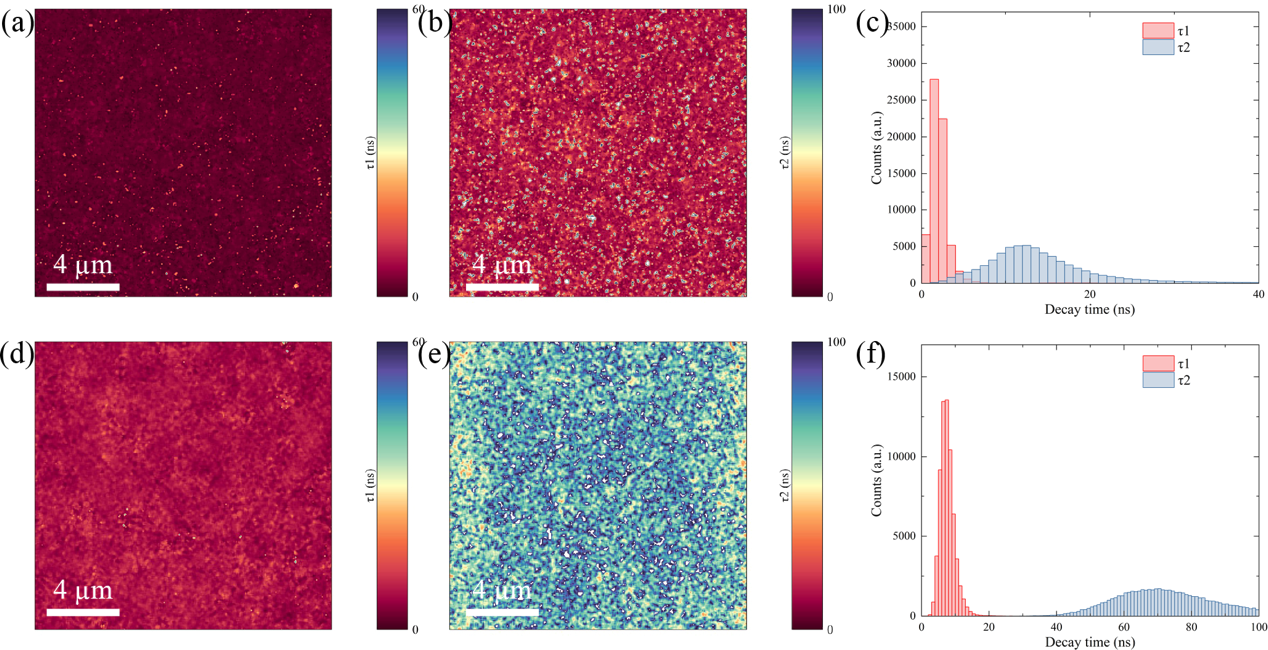


Supplementary Figure 12. Effect of NbO_X_ on carrier lifetimes at the perovskite/C_60_ interface. a) Fast lifetime, b) slow lifetime images, and c) corresponding lifetime distribution histogram of perovskite films before NbO_X_ deposition. d) Fast lifetime, e) slow lifetime images, and f) corresponding lifetime histogram of perovskite films after NbO_X_ deposition.


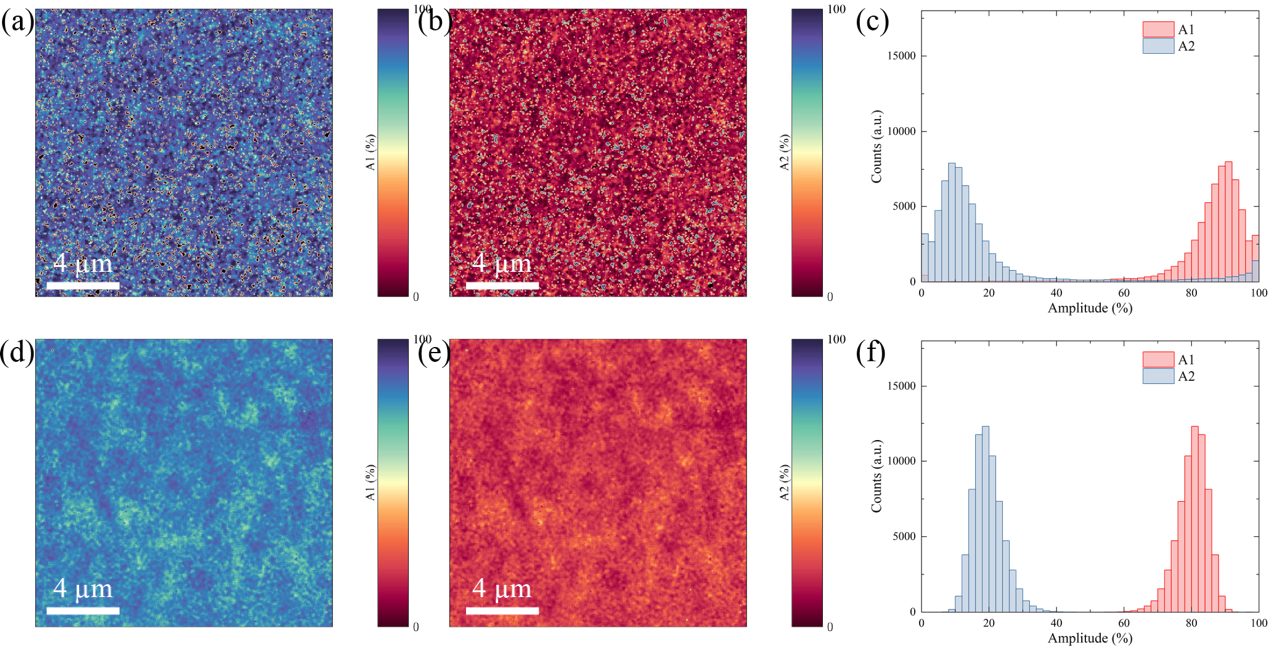


Supplementary Figure 13. Effect of NbO_X_ on the weighting of fast and slow decay components of carriers at the perovskite/C_60_ interface. a) Fast lifetime weight, b) slow lifetime weight images, and c) corresponding lifetime weight distribution histogram of perovskite films before NbO_X_ deposition. d) Fast lifetime weight, e) slow lifetime weight images, and f) corresponding lifetime weight histogram of perovskite films after NbO_X_ deposition.


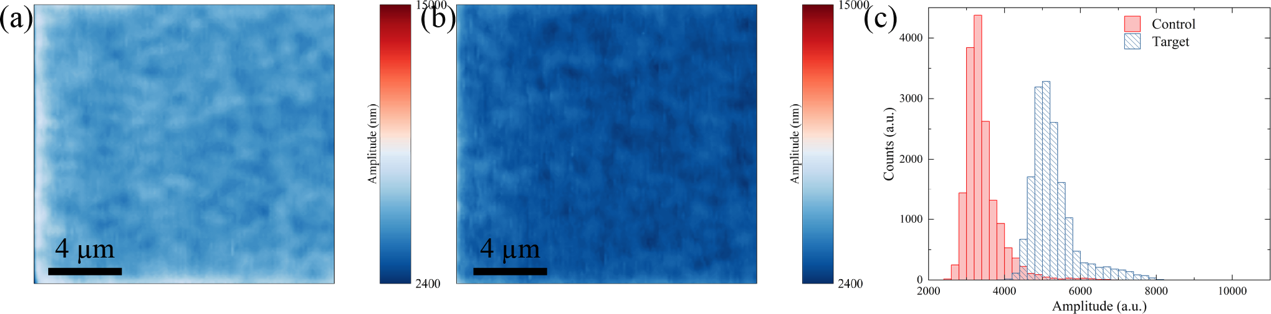


Supplementary Figure 14. Effect of NbO_X_ on the PL spectra at the perovskite/C_60_ interface. PL peak intensity images of perovskite films a) before and b) after NbO_X_ deposition, and c) corresponding PL peak intensity histogram.


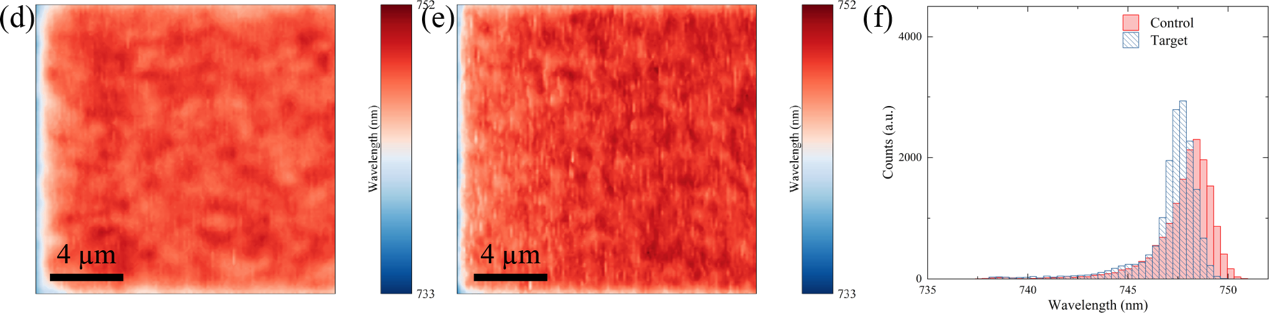


Supplementary Figure 15. Effect of NbO_X_ on the PL spectra at the perovskite/C_60_ interface. PL peak wavelength images a) before and b) after NbO_X_ deposition, and f) corresponding peak wavelength histogram.


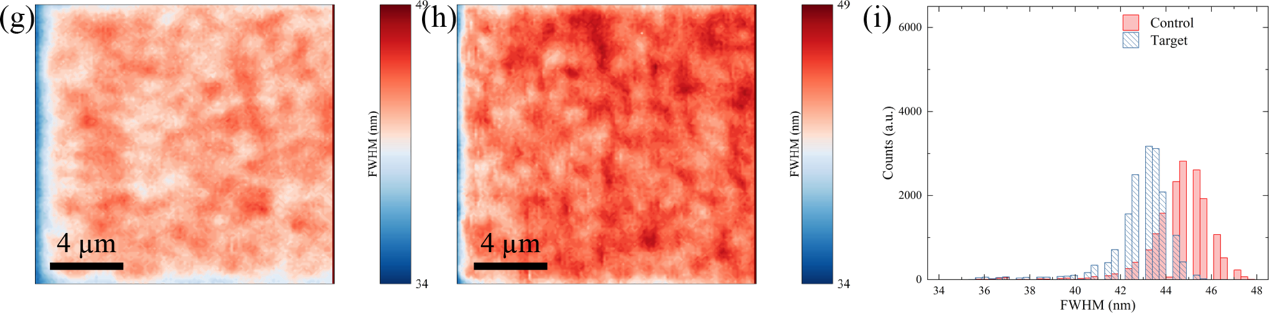


Supplementary Figure 16. Effect of NbO_X_ on the PL spectra at the perovskite/C_60_ interface. PL peak full width at half maximum images a) before and b) after NbO_X_ deposition, and c) corresponding FWHM histogram.


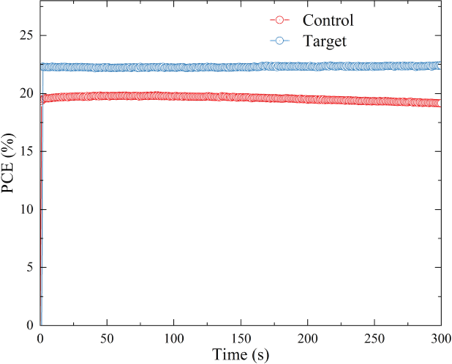


Supplementary Figure 17. The stabilized power output of the control and target single-junction perovskite solar cell.


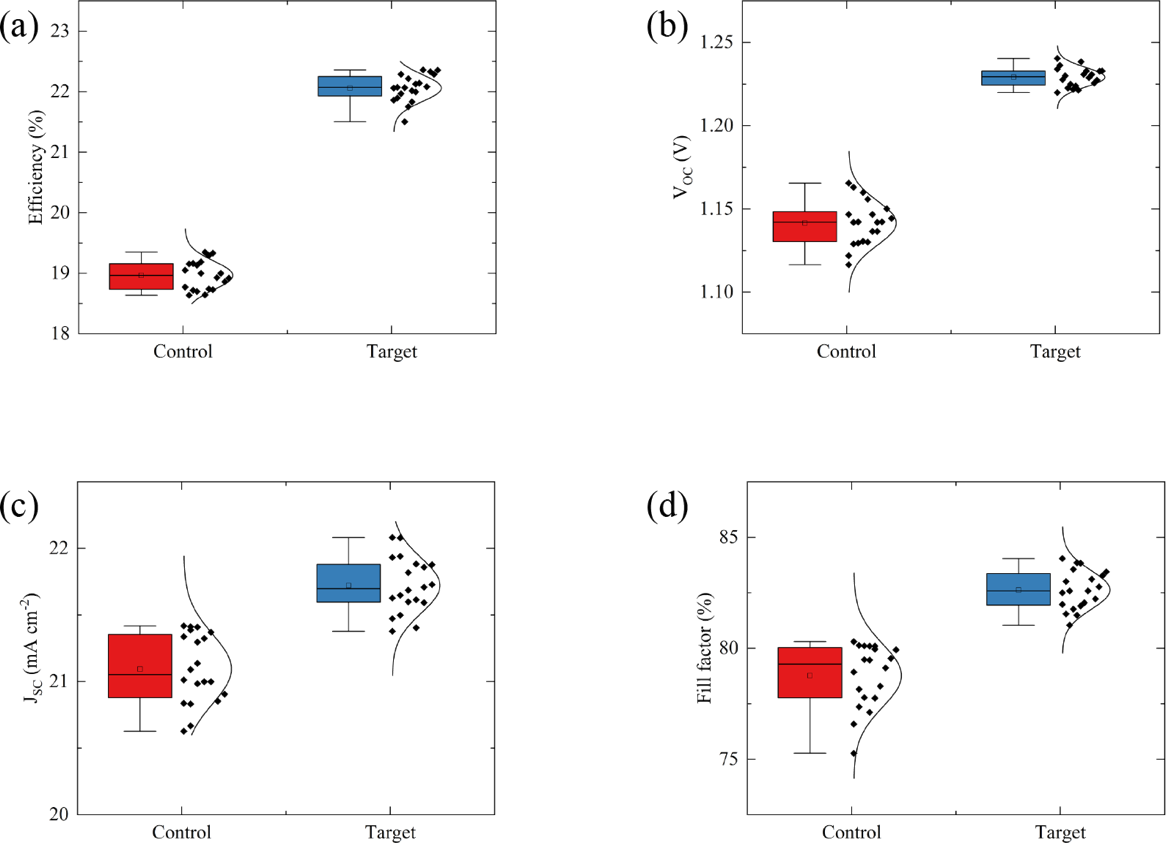


Supplementary Figure 18. Statistics of photovoltaic parameters of single-junction devices. ad, Performance parameters distribution for the control and target single-junction devices: a) PCE, b) V_OC_, c) J_SC_, and d) FF. The box plot denotes median (center line), 75th (top edge of the box), 25th (bottom edge of the box) percentiles. Each experimental condition comprised 20 individual samples for control and target devices. The diamond and curves are the statistical data points and corresponding normal distribution curves. All these performance parameters are obtained on the reverse scan.


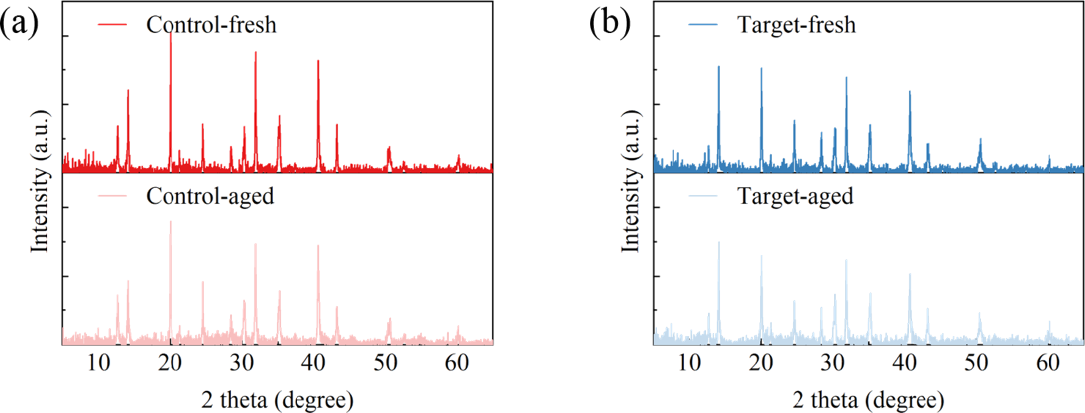


Supplementary Figure 19. Effect of NbO_X_ on the stability of perovskite films. In-situ XRD patterns of the a) control and b) target samples at 85 °C over time. The control shows a pronounced growth of the PbI_2_ characteristic peak at 12.7°, whereas the target sample maintains a stable main perovskite peak at 14.1° with negligible PbI_2_ formation.


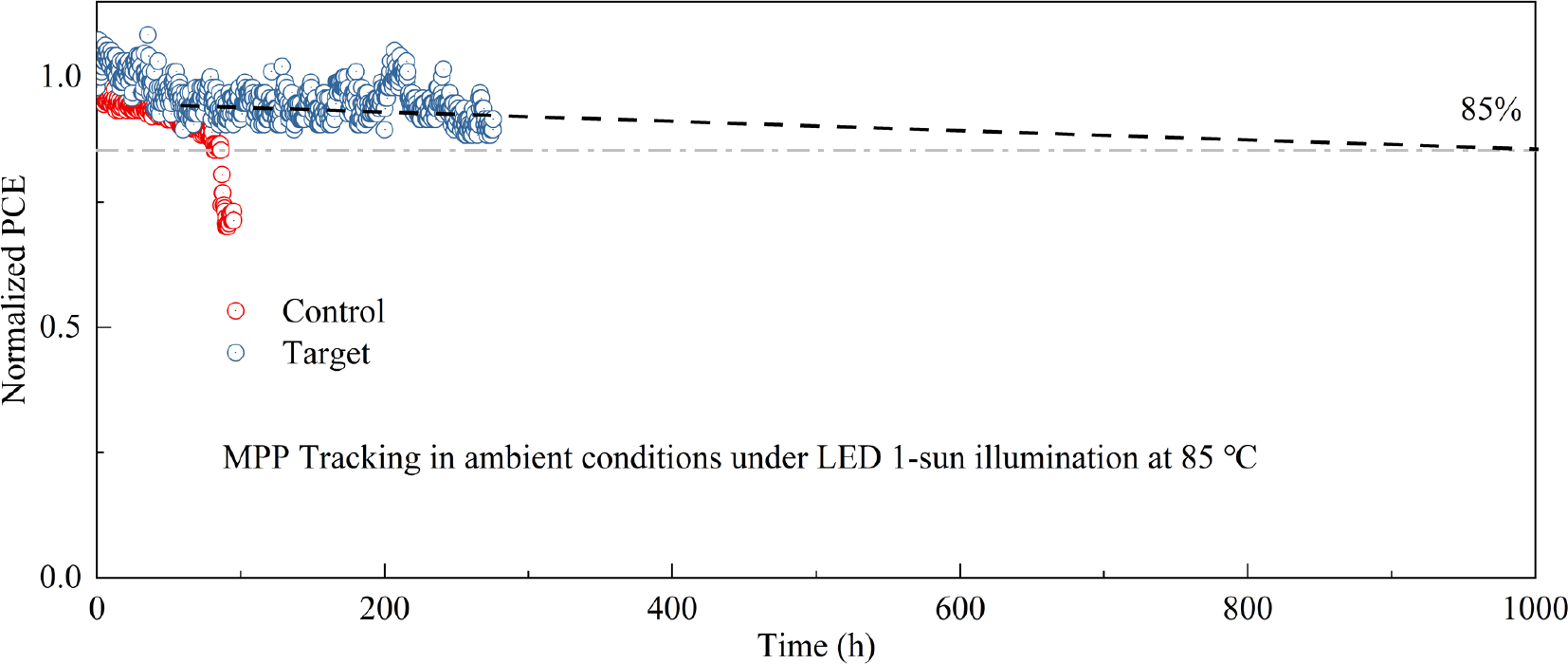


Supplementary Figure 20. Photothermal stability of perovskite single-junction solar cells with and without NbO_X_ passivation.


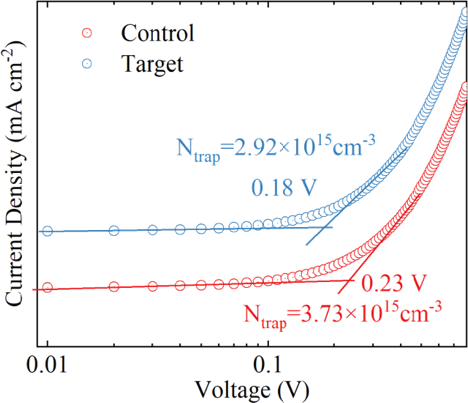


Supplementary Figure 21. Effect of NbO_X_ on the defect density in perovskite single-junction solar cells. Electron defect density of control and target devices measured by the space-charge-limited current method.


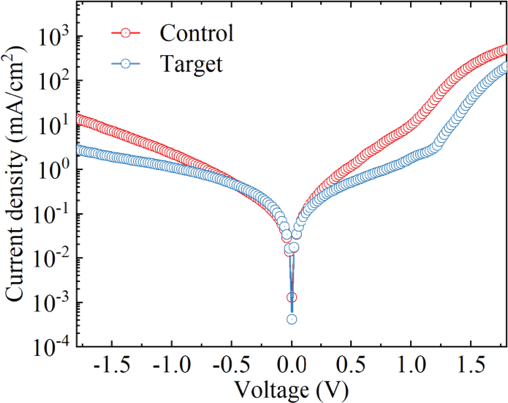


Supplementary Figure 22. Effect of NbO_X_ on the defect density in perovskite single-junction solar cells. Dark J–V curves of control and target devices.


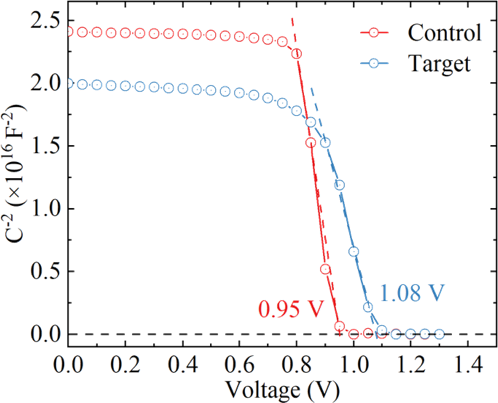


Supplementary Figure 23. Effect of NbO_X_ on the optoelectronic performance of perovskite single-junction solar cells. Capacitance–voltage measurements of control and target devices, with device built-in potential extracted using the Mott–Schottky equation.


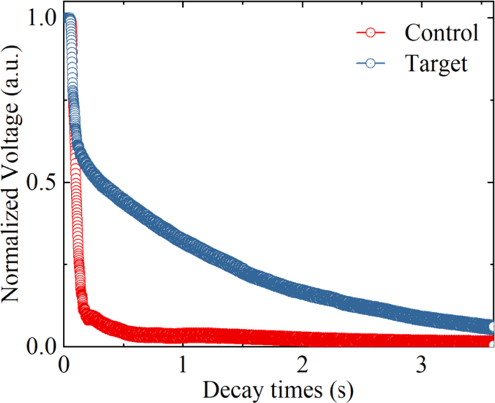


Supplementary Figure 24. Effect of NbO_X_ on the optoelectronic performance of perovskite single-junction solar cells. Transient photovoltage decay curves for evaluating device recombination kinetics and carrier lifetime changes.


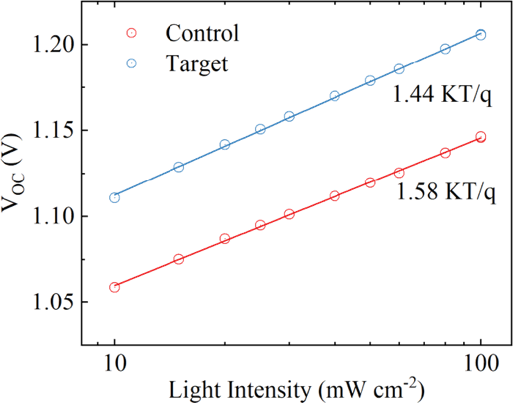


Supplementary Figure 25. Effect of NbO_X_ on the nonradiative recombination behavior of perovskite single-junction solar cells. Open-circuit voltage response curves of different devices under varying light intensities.


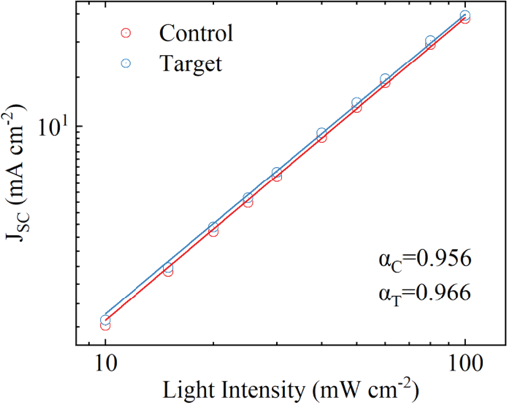


Supplementary Figure 26. Effect of NbO_X_ on the nonradiative recombination behavior of perovskite single-junction solar cells. Power-law fitting of short-circuit current density versus light intensity.


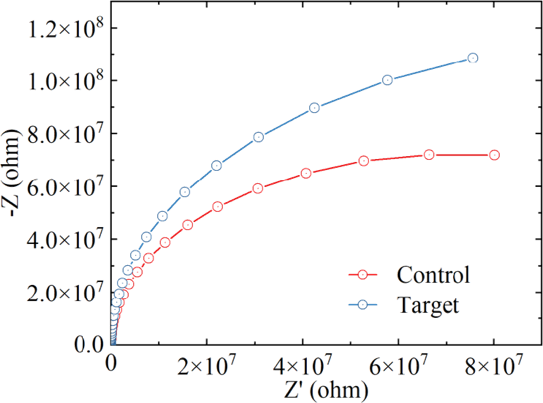


Supplementary Figure 27. Effect of NbO_X_ on the nonradiative recombination behavior of perovskite single-junction solar cells. Electrochemical impedance spectra of the control and target devices.


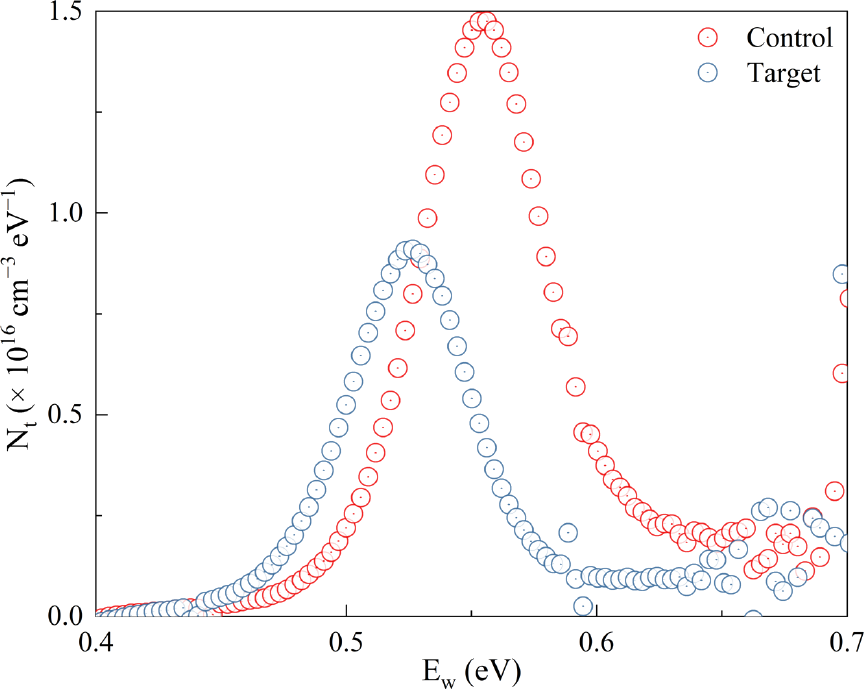


Supplementary Figure 28. Trap density of states (tDOS) results for perovskite films with and without NbO_X_.


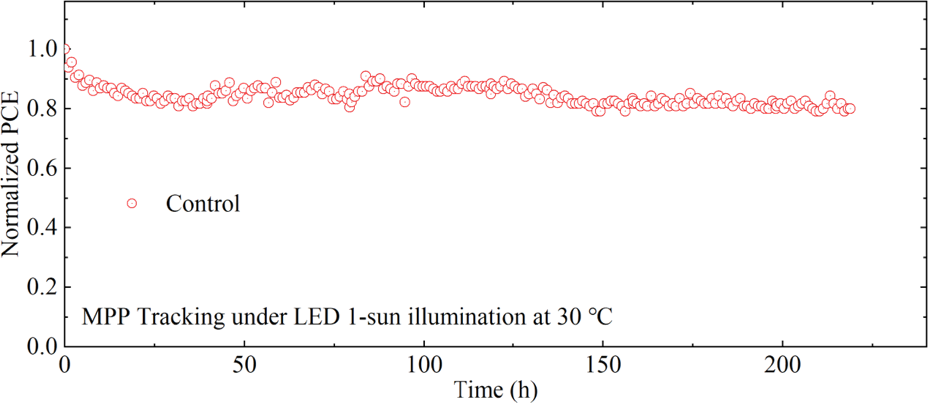


Supplementary Figure 29. Operational stability of control perovskite/silicon tandem solar cells.

**Supplementary Table 1. Effect of the NbO_X_ film on the full width at half maximum (FWHM) of the perovskite (100) peak and the PbI₂/(100) peak intensity ratio (extracted from XRD data).**

| Samples | FWHM (°) | Area ratio |
| --- | --- | --- |
| Control | 0.135 | 0.539 |
| Target | 0.122 | 0.405 |

**Supplementary Table 2. Fitting parameters of TRPL decay curves for different perovskite films.**

| Samples | τ₁ (ns) | A_1_ (%) | τ_2_ (ns) | A_2_ (%) | τ_eff_ (µs) |
| --- | --- | --- | --- | --- | --- |
| Control | 5.349 | 88.53 | 38.589 | 11.47 | 0.916 |
| Target | 9.017 | 82.95 | 102.562 | 17.05 | 2.495 |

**Supplementary Table 3. Fitting parameters of TRPL decay curves for different perovskite films with C_60_ cappping layer.**

| Samples | τ₁ (ns) | A_1_ (%) | τ_2_ (ns) | A_2_ (%) | τ_eff_ (µs) |
| --- | --- | --- | --- | --- | --- |
| Control | 2.732 | 91.88 | 22.790 | 8.12 | 0.436 |
| Target | 7.609 | 89.22 | 94.299 | 10.78 | 1.695 |

**Supplementary Table 4. Photovoltaic performance parameters of perovskite single-junction devices with varying NbO_X_ thicknesses.**

|  | Voc (V) | Jsc (mA cm^-2^) | FF (%) | PCE (%) | R_s_ (ohm) |
| --- | --- | --- | --- | --- | --- |
| Control | 1.15 | 21.09 | 79.90 | 19.32 | 58.43 |
| 1 nm-NbO_X_ | 1.19 | 21.45 | 81.01 | 20.70 | 52.62 |
| 2 nm-NbO_X_ | 1.23 | 21.73 | 83.45 | 22.37 | 50.63 |
| 5 nm-NbO_X_ | 1.19 | 21.33 | 80.43 | 20.40 | 60.27 |
| 10 nm-NbO_X_ | 1.16 | 21.30 | 77.82 | 19.21 | 63.58 |

**Supplementary Table 5. Photovoltaic parameters of perovskite single-junction devices before and after NbO_X_ passivation.**

| Samples | | V_OC_ (V) | J_SC_ (mA·cm^–2^) | FF (%) | PCE (%) | HI (%) |
| --- | --- | --- | --- | --- | --- | --- |
| Control | Forward | 1.14 | 21.1 | 79.5 | 19.2 | 0.5 |
|  | Reverse | 1.15 | 21.1 | 79.9 | 19.3 |  |
| Target | Forward | 1.23 | 21.7 | 83.3 | 22.3 | 0.4 |
|  | Reverse | 1.23 | 21.7 | 83.4 | 22.4 |  |

**Supplementary Table 6. Photovoltaic parameters of perovskite/TOPCon tandem solar cells with NbO_X_ interlayer.**

| Samples | V_OC_ (V) | J_SC_ (mA·cm^–2^) | FF (%) | PCE (%) |
| --- | --- | --- | --- | --- |
| Forward | 1.92 | 20.5 | 78.9 | 31.1 |
| Reverse | 1.93 | 20.5 | 80.6 | 32.0 |
